# Composition and activity of nitrifier communities in soil are unresponsive to elevated temperature and CO_2_, but strongly affected by drought

**DOI:** 10.1038/s41396-020-00735-7

**Published:** 2020-08-07

**Authors:** Joana Séneca, Petra Pjevac, Alberto Canarini, Craig W. Herbold, Christos Zioutis, Marlies Dietrich, Eva Simon, Judith Prommer, Michael Bahn, Erich M. Pötsch, Michael Wagner, Wolfgang Wanek, Andreas Richter

**Affiliations:** 1 Centre for Microbiology and Environmental Systems Science, University of Vienna, Althanstrasse 14, 1090 Vienna, Austria; 2 Joint Microbiome Facility of the Medical University of Vienna and the University of Vienna, Vienna, Austria; 3 Department of Ecology, University of Innsbruck, 6020 Innsbruck, Austria; 4 Agricultural Research and Education Centre Raumberg-Gumpenstein, Altirdning 11, 8952 Irdning, Austria; 5 Department of Chemistry and Bioscience, Aalborg University, Aalborg, Denmark; 6 International Institute for Applied Systems Analysis, Laxenburg, Austria

**Keywords:** Climate-change ecology, Biogeochemistry, Microbial ecology

## Abstract

Nitrification is a fundamental process in terrestrial nitrogen cycling. However, detailed information on how climate change affects the structure of nitrifier communities is lacking, specifically from experiments in which multiple climate change factors are manipulated simultaneously. Consequently, our ability to predict how soil nitrogen (N) cycling will change in a future climate is limited. We conducted a field experiment in a managed grassland and simultaneously tested the effects of elevated atmospheric CO_2_, temperature, and drought on the abundance of active ammonia-oxidizing bacteria (AOB) and archaea (AOA), comammox (CMX) *Nitrospira*, and nitrite-oxidizing bacteria (NOB), and on gross mineralization and nitrification rates. We found that N transformation processes, as well as gene and transcript abundances, and nitrifier community composition were remarkably resistant to individual and interactive effects of elevated CO_2_ and temperature. During drought however, process rates were increased or at least maintained. At the same time, the abundance of active AOB increased probably due to higher NH_4_^+^ availability. Both, AOA and comammox *Nitrospira* decreased in response to drought and the active community composition of AOA and NOB was also significantly affected. In summary, our findings suggest that warming and elevated CO_2_ have only minor effects on nitrifier communities and soil biogeochemical variables in managed grasslands, whereas drought favors AOB and increases nitrification rates. This highlights the overriding importance of drought as a global change driver impacting on soil microbial community structure and its consequences for N cycling.

## Introduction

Nitrogen (N) cycling is a fundamental process in terrestrial ecosystems, critical for the functioning of all living organisms. Soil ecosystems are currently undergoing substantial changes, as a consequence of anthropogenic activities [1, 2]. Microorganisms catalyze most soil N transformation processes, and thus play a central role in mediating N exchange between the atmosphere, plants, and soils [3]. Thus, understanding the nature and the intensity of the responses of soil microorganisms to climate change is of utmost importance to predict the future terrestrial N cycle, including its repercussion on plant productivity and climate-carbon feedbacks [3, 4]. Yet, our understanding of the relative role of specific microbial taxa in mediating N transformations in terrestrial environments in response to climate change is still incomplete. In spite of current climate projections [5] the interactive effects of elevated temperature, elevated atmospheric CO_2_, and changes in precipitation patterns have rarely been assessed together with regards to microbially mediated soil N transformations [6, 7].

Nitrification represents a key process in the terrestrial N cycle. It has long been considered to be a two-step process initiated with the oxidation of ammonia to NO_2_^−^ by ammonia-oxidizing bacteria (AOB) and ammonia-oxidizing archaea (AOA), followed by NO_2_^−^ oxidation to NO_3_^−^ by nitrite-oxidizing bacteria (NOB) [8]. Based on metagenomic surveys, marker gene assays and functional gene studies targeting the *amoA gene*, (gene that codes for the alpha subunit of ammonia monooxygenase) several studies demonstrated the influence of different environmental factors on the abundance and composition of nitrifying communities, most notably substrate concentration, soil water content and soil pH [9–11]. Furthermore, all known ammonia-oxidizers contribute to nitric (NO) and nitrous (N_2_O) oxide emissions, but the yield by which they do differs strongly between groups, with AOB showing the highest N_2_O yield per mol of oxidized ammonia when compared with other groups [12]. Several studies have reported a numeric dominance of AOB over AOA in agricultural and grassland soils to which fertilizers are applied on a regular basis, leading to the idea that AOB are responsible for the ammonia-oxidation step in these environments [13, 14]. In contrast, AOA were thought to be the main ammonia-oxidizing taxa in N poor environments, as well as in acidic soils [15]. Notably, all known AOB have a relatively low substrate affinity, whereas AOA encompass members with a broad range of substrate affinities [12]. NOB span six genera, out of which *Nitrospira* is the most phylogenetically diverse and widespread across different habitat types including terrestrial ecosystems [16]. Recently, it was discovered that some members of this genus (Comammox *Nitrospira*; CMX) can catalyze both steps of nitrification [17, 18]. Although our knowledge on the distribution and diversity of comammox *Nitrospira* is still comparatively limited, several studies have reported their presence and activity in terrestrial environments [19, 20]. Furthermore, it has been demonstrated that the only cultured comammox species *N. inopinata*, has a high substrate affinity [12]. Thus, a joint assessment of the relative contribution of the aforementioned microbial groups to nitrification is needed, as well as of their specific responses to climate change.

Current global change in temperate ecosystems is characterized, amongst other changes, by rising air and soil temperature, elevated CO_2_ concentrations, and changes in water availability, such as increased frequencies of drought events. Elevated atmospheric CO_2_ concentrations affect soil microorganisms by promoting plant growth and photosynthetic activity [21]. This can result in increased plant belowground C allocation and rhizodeposition [22, 23], which stimulates heterotrophic microbial activity and mobilizes N. However, higher plant growth can indirectly affect soil microorganisms by gradually depleting the soil N pool available for microbial uptake [24, 25], potentially causing microorganisms to become N limited. Previous studies revealed a decrease in the relative abundance of AOA and AOB in response to elevated CO_2_ [26, 27]. In contrast to elevated CO_2_, elevated temperature directly accelerates microbial processes and turnover rates [28], alters water and nutrient availability, extends the plant growing season [29] and may favor the growth of slow growing bacteria [30]. Concomitantly, studies have reported significant changes in *amoA* gene abundance and expression of AOA and AOB in response to temperature [27, 31]. The growth, activity, and community structure of AOA, but not of AOB have been shown to change in response to elevated temperature in soil mesocosms [31]. On the other hand, a comprehensive study across 23 different soil types has highlighted temperature as the most important factor affecting AOB community structure [32]. Finally, extreme events such as droughts can cause soil microorganisms to decrease their activity levels, to accumulate osmoprotectants/compatible solutes, and often to switch to dormancy [33]. A study in mountain grasslands showed that drought had distinct effects on the relative abundance of ammonia-oxidizing microorganisms, with reduced AOA *amoA* abundance with drought and no observed changes in AOB *amoA* abundance [11].

Multifactorial studies that particularly target nitrifier communities are scarce, and most studies are rather focused on the response of broad ecosystem N processes to global change [34–36].

Therefore, the goal of this study was to understand how elevated atmospheric CO_2_ (eCO_2_), elevated temperature (eT) and drought (D), alone or in combination, affect the relative abundance and diversity of nitrifier communities and their activity (i.e., gross nitrification rates) in a managed submontane grassland. We expected to observe: (i) a shift in active nitrifier community structure towards high affinity nitrifiers (AOA and CMX) as a consequence of lower NH_4_^+^ availability at eCO_2_; (ii) an overall increase in nitrification rates and *amoA* expression levels of AOA, AOB, and potentially CMX in response to eT; and (iii) a reduction of the abundance and activity of all nitrifying groups by drought. Our study also intended to explore whether the combined effect of eCO_2_ and eT—with or without drought—would have additive or nonadditive effects on the diversity and abundance of nitrifiers.

## Methods

### Site description and experimental design

The experimental site is located at the agricultural research institute HBLFA Raumberg‐Gumpenstein in Styria, Austria (47°29′38″N, 14°06′03″E) and is part of a long-term climate change experiment (ClimGrass). With a mean annual temperature of 8.2 °C and a mean annual precipitation of 1056 mm, this area is representative of a large part of alpine grasslands. The grassland under study was established in 2007 and is dominated by the grasses *Arrhenatherum elatius* and *Festuca pratensis* together with legume species such as *Lotus corniculatus* and *Trifolium pratense*. It also includes a few subdominant non‐leguminous forbs such as *Taraxacum officinale and Plantago lanceolata*. The soil type is a Cambisol (WRB classification) with loamy sand texture and a pH of 5.

The experimental design was originally based on a response surface regression approach that aimed to study the nature and intensity of the grassland’s responses to manipulations of elevated atmospheric temperature and CO_2_, and drought [37]. The experimental site comprised a total of 54 plots showing individual and combined effects of three levels of temperature (+1.5 °C, + 3 °C), atmospheric CO_2_ concentrations (ambient, +150 ppm, +300 ppm) and drought (Fig. S1). A detailed explanation regarding the reasons behind the choice of treatments and corresponding number of replicates can be found in [37]. Each treatment had a different number of replicates, which reflected a balance between statistical power and budget constraints [37].

For this specific experiment, we selected only a set of soil samples from 26 plots (4 × 4 m each) during peak growing season in July 2017 (Table 1; Fig. S1). Treatments included soil plots on which temperature (eT; +3 °C) and CO_2_ (eCO_2_; +300 ppm CO_2_) were manipulated individually or in combination, as well as plots on which drought (D) was simulated through the use of automatic rainout shelters installed 8 weeks before sampling (Table 1; Fig. S1). Manipulations in atmospheric CO_2_ concentrations were done through a miniFACE—free air CO_2_ enrichment system, whereas atmospheric temperature was manipulated through the use of infrared heaters. Since 2014, infrared heaters were continuously on except when snow cover exceeded a depth of 10 cm. The soil temperatures measured by soil probes in selected plots between June and July 2017 showed an average of +1.86 °C for plots affected by elevated temperature, compared with the controls (data not shown). CO_2_ fumigation was only applied during the growing season (April to November). Site management included mowing (three times a year) and mineral fertilization (90 kg N ha^−1^ a^−1^ as NH_4_NO_3_, 65 kg P ha^−1^ a^−1^ as Ca(H_2_PO_4_)^2^ and 170 kg K ha^−1^ a^−1^ as K_2_O), which corresponds approximately to the amount of NPK removed by biomass harvest, in order to replicate local farming practices.

**Table 1:** Soil plots sampled in this study.

	Treatments used for statistical analyses		
Treatment	Description	Individual and combined effects of temperature and CO_2_	Individual and combined effects of future climate and drought
[Amb]	Ambient conditions (*n* = 8)	×	×
[eT]	+3 °C above ambient (*n* = 3)	×	
[eCO_2_]	+300 ppm CO_2_ above ambient (*n* = 3)	×	
[eT] × [eCO_2_]	+3 °C and +300 ppm CO_2_ above ambient (*n* = 4)	×	×
[Amb] × [D]	Ambient conditions + rainout shelter (*n* = 4)		×
[eTeCO_2_] × [D]	+3 °C and +300 ppm CO_2_ above ambient + rainout shelter (*n* = 4)		×

Biological replicates are shown in parenthesis. Specific soil plots were selected in order to test for either the individual and combined effects of elevated temperature and CO_2_ or the individual and combined effects of future climate and drought. This separation was done in order to have a full factorial design for each set. The spatial arrangement of all the plots is shown in Fig. S1.

### Sample collection and soil physicochemical analyses

Immediately after removal of aboveground biomass several (4–11) soil cores were taken per plot to a depth of 10 cm using a stainless-steel soil corer with 2 cm inner diameter. Stones and roots were picked, washed, dried and weighed, and soils were sieved through a 2 mm sieve directly after collection. Fresh soil subsamples for molecular analyses were immediately frozen in liquid N. The remaining fresh soil was transported to the laboratory and incubated shortly (2–3 days) at the respective in situ temperatures measured at the time of collection (17 °C for ambient temperature plots and 20 °C for elevated temperature plots) until analysis.

Gravimetric soil water content was determined by oven drying (105 °C for 24 h) 5 g of fresh, sieved soil. Dissolved soil organic C (DOC), nitrogen (DON) and total dissolved N (TDN) were measured in 1 M KCl extracts (using a soil to solution ratio of 1:7.5 w–v) after filtering through ash-free cellulose filters (Whatman, GE Healthcare Life Sciences) on a TOC/TN analyzer (TOC-VCPH/TNM-1, Shimadzu, Austria). In parallel, soils were chloroform fumigated for 48 h in order to estimate microbial biomass nitrogen (MBN) [38]. After fumigation, soils were also extracted with 1 M KCl, and MBN was calculated as the difference in TDN between fumigated and non-fumigated soils. Ammonium (NH_4_^+^) and nitrate (NO_3_^−^) concentrations were determined photometrically in the 1 M KCl extracts of non-fumigated soils [39]. Soil pH was determined in a 1:5 mix of dry soil and 0.01 M CaCl_2_ solution with a pH meter (Sentron Europe BV).

### Isotope pool dilutions

Gross rates of N mineralization, and nitrification were determined and calculated from fresh, sieved soil samples using ^15^N pool dilution assays [40].

Two to three grams of duplicate soil samples were incubated with ^15^NH_4_Cl and K^15^NO_3_ (98 at%) tracer solutions for 4 and 24 h at the corresponding in situ field temperatures. We aimed to increase the ^15^N enrichment of the target pool to ~20 at%^15^N and added 100 µl of tracer solution per gram of fresh soil, after a previous photometrical determination of background NH_4_^+^ and NO_3_^−^pool sizes. The addition of liquid the tracer increased the soil water in ambient and drought plots by 1.79 and 4.56-fold respectively. Incubations were stopped after 4 and 24 h by the addition of 1 M KCl (1:7.5 w–v), extraction by horizontal shaking on an orbital shaker for 30 min (150 rpm) and filtration through ash-free cellulose filters. Mineralization extracts were prepared using a microdiffusion method [41] followed by the measurement of ^15^/^14^N isotope ratios and concentrations of NH_4_^+^ in the acid traps by elemental analyzer-isotope ratio mass spectrometry (EA-IRMS; EA1110 coupled via ConFlo III interface to a Delta^PLUS^ IRMS, Thermo Fisher, Bremen, DE [42]. Concentrations and ^15^/^14^N isotope ratios of NO_3_^−^ in 1 M KCl extracts were determined by converting NO_3_^−^ to NO_2_^−^ with vanadium (III) chloride (VCl_3_) and further reduction of NO_2_^−^ to N_2_O by sodium azide (NaN_3_) [42]. Concentrations and ^14^/^15^N isotope ratios of the resulting N_2_O were determined by purge-and-trap isotope ratio mass spectrometry (PT-IRMS), using a Gasbench II headspace analyzer (Thermo Fisher, Bremen, DE) with a cryo-focusing unit, coupled to a Finnigan Delta V Advantage IRMS (Thermo Fisher, Bremen, DE).

### TNA extraction, RNA purification, and cDNA synthesis

Total nucleic acids (TNA) were extracted from 0.4 g frozen soil by bead-beating in the presence of a cetyl trimethylammonium bromide buffer and phenol, according to a previously published extraction protocol [43] and eluted in 250 µl of Low-Tris-EDTA buffer. Following extraction, samples were purified using the OneStep^TM^ PCR Inhibitor Removal Kit (Zymo, Irvine, CA, USA) and TNA was quantified using the Quant-iT^TM^ PicoGreen® Kit (Thermo Fisher Scientific), according to the manufacturers’ instructions. DNA was digested in 1–3 µg of TNA extract with Turbo DNase (Thermo Fisher Scientific), and RNA was purified using the GeneJET RNA Cleanup and Concentration Micro Kit (Thermo Fisher Scientific) and eluted in 25 µl of RNase Storage Solution (Thermo Fisher Scientific). The RNA yield was quantified using the Quant-iT^TM^ RiboGreen^®^ assay (Thermo Fisher Scientific). Successful DNA digestion was confirmed by negative results on a gel electrophoresis after a DNA-targeted SSU rRNA gene PCR assay on the purified RNA extracts using the primer pair 515F-mod/806R-mod [44, 45]. Afterwards, cDNA was synthesized from 400 ng of RNA extract using random hexamer primers and SuperScript^TM^ IV reverse transcriptase (Thermo Fisher Scientific) following the manufacturer´s instructions and eluted in 54 µl of nuclease-free water.

### amoA/nxrB gene and transcript amplification and sequencing

The diversity of the ammonia-oxidizing microbial community, as well as the NOB community was assessed via amplification and sequencing of the ammonia monooxygenase enzyme subunit A (*amoA*) gene, and the nitrite oxidoreductase subunit beta (*nxrB*) gene respectively, with functional group specific primers modified to include a linker sequence (‘head’) for barcoding PCRs at the 5′ end, as described previously [46]. Detailed amplification conditions can be found in the Supplementary Material and Methods file. Library preparation and sequencing services were provided by Microsynth (Balgach, Switzerland). The library was prepared by adapter ligation and PCR using the TruSeq Nano DNA Library Prep Kit according to the TruSeq nano protocol (Illumina, FC-121–4003), but excluding the fragmentation step. Sequencing was performed on a MiSeq platform, v3, 2 × 300 bp (Illumina). Paired MiSeq reads were processed according to [46]. AOB, and AOA *amoA* gene OTUs were clustered at 95% and 96% sequence identity, respectively [47, 48]. A sequence identity cutoff of 95% was used for NOB *nxrB* OTUs and for CMX clade B *amoA* OTUs. Taxonomic classification of AOA, AOB-related (*Nitrosomonas*), and NOB OTUs was performed using the evolutionary placement algorithm implemented in RAxML to place OTUs into a reference tree using a set of classified full-length nucleotide sequences [49, 50]. Uncultured *Nitrosospira* (AOB) *amoA* OTUs were classified by local nucleotide BLAST (version 2.8.1+) searches against a *Nitrosospira* reference database [51] since phylogenetic trees among reference *Nitrosospira* showed no stable tree topology for mapping reads. Represented sequences for all CMX clade B *amoA* were aligned against a reference *pmoA* and *amoA* database [52] using the SINA aligner [53] and a maximum likelihood phylogenetic tree was calculated based on this alignment using W-IQ-Tree [54] with 1000 bootstrap iterations and ModelFinder to determine the best fitting base substitution model [55]. The resulting tree was visualized with iTOL [56].

All sequence alignments were performed with Mafft [57] and manually inspected. OTUs with no similarity to the functional group of interest were considered unspecific co-amplification and excluded from further analysis (Table S1).

### AmoA gene and transcript quantification by qPCR

Gene and transcript copy numbers of *amoA* genes of betaproteobacterial AOB, AOA, and CMX clade B were determined by qPCR. Gene and transcript copy numbers of *Nitrospira nxrB* were not assessed in this study due to the highly variable gene copy number per genome, which would hamper precise quantifications. In addition, the primer pair used for amplicon sequencing co-amplifies several nonspecific amplicons, which would also make quantifications inaccurate [58].

Assays with different sample dilutions (5× to 10,000×), resulting in template amounts ranging from 0.004 to 24 ng, were performed to minimize possible PCR inhibition caused by excess template or co-extracted inhibitory substances. For all samples, the highest dilutions that yielded consistent results were used to calculate gene and transcript copy numbers. All assays were performed in triplicate for each dilution. Detailed amplification conditions can be found in the Supplementary Material and Methods file. For each assay, standard series were generated by tenfold serial dilutions (10^8^–10^1^ gene copies μl^−1^) from purified M13-PCR products obtained from environmental samples (CMX clade B *amoA*) or pure cultures of *Nitrosomonas europaea* and *Nitrososcosmicus hydrocola*, as described previously [52, 59]. The average amplification efficiency for the comammox clade B *amoA*, archaeal *amoA*, and betaproteobacterial *amoA* assays was 90.6%, 93.6% and 89,2% respectively.

### Statistical analyses

**Fig. 1:**
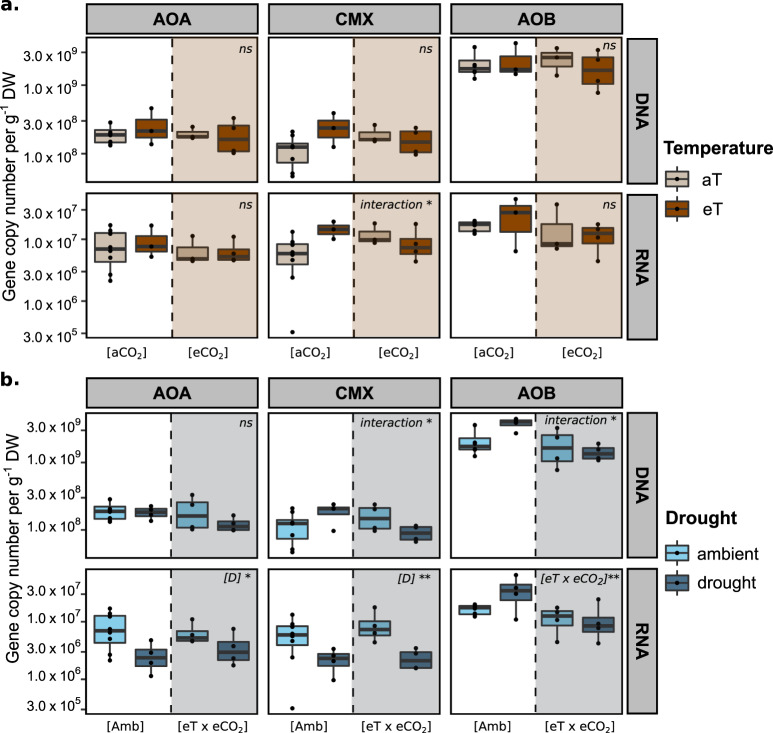
qPCR quantifications of amoA gene and transcript copy numbers per gram of dry soil from the ammonia-oxidizing microbial groups. **a** Quantifications from plots affected by elevated temperature (eT) and atmospheric CO_2_ concentrations (eCO_2_). The dashed lines represent plots affected by elevated atmospheric CO_2_ conditions (eCO_2_). The colors represent plots affected by either ambient temperature (aT) or elevated temperature (eT). **b** Quantifications from plots affected by future climate (eT x eCO_2_) and drought (D). The dashed lines separate plots affected by drought (D). The colors represent the drought treatment. The caption on the upper right corner of each subplot represents the ANOVA results. *p value <0.05; **p value <0.01. Whenever the ANOVA results showed a significant interaction term, lsmeans multiple comparison tests were ran. For details on the ANOVA and post-hoc tests, see Tables S2 and S3.

All statistical analyses were performed in R (version 3.5.1). Statistical analyses were performed with two separate linear models using the *Anova()* function of the package “car” following a full factorial design that allowed to test for interactions between all factors under analysis. This approach was chosen because the drought treatment was imposed on a reduced set of treatments (Table 1; Fig. S1) that did not include soil plots where eT and eCO_2_ were manipulated individually. Therefore, the first model (eT vs. eCO_2_ dataset) included [eCO_2_], [eT] and their interactions. The second model (drought dataset) included drought, [eT × eCO_2_] and their interactions. Both models were used to assess if individual global change effects were modified when variables were combined (interactive effects) in order to reveal potential additive, synergistic or antagonistic effects (Tables 2 and 3, Fig. 1, Table S2). Model assumptions of normality and homoscedasticity were checked on the model residuals [60] and variables were transformed when needed to meet model assumptions. Nonparametric tests were conducted in case assumptions were not met after data transformation. Multiple comparisons were performed on the interactive effects using the Least Square Means within the R package “lsmeans” (Table S3). In order to account for the potential effect of different treatment replicates, we used a type II ANOVA, except whenever an interaction effect was observed and a type III ANOVA was used instead [61]. Sequencing data were analyzed with the “phyloseq” package [62]. The Shannon diversity index was calculated after removing OTUs not observed more than three times in at least 10% of the samples. Specific sequencing barcodes per sample, and the number of reads that passed the filtering criteria can be found in the Supplementary Datafile 1. We used a permutational analysis of variance (PERMANOVA) with 9999 permutations on a dissimilarity matrix (Morisita–Horn on top of a Hellinger transformed OTU table) to assess the effect of the variables under study on functional gene diversity using the function *adonis()* in the “vegan” package with a two-factorial design [63] (Table S4). Similarly to the ANOVA analyses, we developed two models, the first model (eT vs. eCO_2_ dataset) included [eCO_2_], [eT], and their interactions, the second model (drought dataset) included drought, [eT × eCO_2_] and their interactions. This test is sensitive to dispersions among groups, which might be more pronounced in situations where there is a different number of replicates per treatment and thus confound significant PERMANOVA results. Therefore, we confirmed *a priori* that groups did not differ significantly in their dispersion by performing an analysis of multivariate homogeneity (PERMDIST) using the function *betadisper()* of the package ‘vegan’, with the bias.adjust=T argument, to account for differences in sample size [64, 65]. All raw sequencing data were deposited into the NCBI SRA under BioProject ID PRJNA612521. All the remaining data are available as Supplementary Material.

**Table 2:** Average values for soil parameters, organic and inorganic pools and process rates (means ± SE).

	Mean values per treatment											
	[Ambient]		[eT]		[eCO_2_]		[eT] × [eCO_2_] [eT × eCO_2_]		[Ambient] × [D]		[eT × eCO_2_] × [D]	
	Mean ± SE		Mean ± SE		Mean ± SE		Mean ± SE		Mean ± SE		Mean ± SE	
	(*n* = 8)		(*n* = 3)		(*n* = 3)		(*n* = 4)		(*n* = 4)		(*n* = 4)	
Soil parameters												
SWC	0.34	0.01	0.32	0.01	0.34	0.00	0.33	0.01	0.08	0.01	0.06	0.01
pH	4.95	0.03	5.02	0.05	4.99	0.03	4.97	0.03	4.96	0.04	5.01	0.02
Soil N	0.31	0.01	0.27	0.02	0.26	0.02	0.29	0.02	0.32	0.01	0.33	0.03
MBN	79.31	5.89	93.39	14.16	63.65	9.89	93.34	6.59	99.44	7.09	120.26	6.03
Extractable organic and inorganic pools												
NO_3_^−^	2.97	0.64	5.18	2.30	4.19	1.47	5.95	2.46	5.98	1.72	5.26	1.09
NH_4_^+^	1.11	0.20	0.87	0.12	0.95	0.13	0.89	0.11	12.23	4.54	13.13	2.99
DIN	4.09	0.76	6.04	2.25	5.13	1.45	6.84	2.51	18.21	6.15	18.39	3.56
DON	14.54	2.21	8.51	1.90	12.37	0.67	12.65	1.83	19.34	1.18	23.78	3.45
TDN	18.63	2.78	8.51	1.91	12.37	0.68	19.49	1.07	37.55	7.08	42.16	4.48
DOC	87.28	8.65	93.79	3.26	79.99	0.88	94.07	7.93	165.68	45.37	215.09	59.17
Gross process rates												
NH_4_^+^ mineralization^a^	2.72	0.38	1.75	0.76	2.62	0.35	2.97	0.49	4.03	0.30	6.97	0.81
NH_4_^+^ immobilization^ab^	2.08	0.49	1.14	0.51	1.82	0.23	3.44	1.05	5.18	1.73	10.59	3.89
Nitrification	1.77	0.42	2.24	0.80	2.73	0.20	2.38	0.73	3.89	0.17	4.56	0.62

The number of biological replicates is shown in parenthesis. Soil water content (SWC) is expressed in % of fresh soil; *MBN* microbial biomass N and *TDN* total dissolved nitrogen are expressed in µg N g^−1^ DW; *DIN* dissolved inorganic nitrogen and *DON* dissolved organic nitrogen are expressed in µg N g^−1^ DW. Ammonium (NH_4_^+^) and nitrate (NO_3_^−^) are expressed in µg N g^−1^ DW. *DOC* dissolved organic carbon is expressed in µg C g^−1^ DW. All process rates are expressed in µg N g^−1^ DW d^−1^.

^a^*n* = 3 for treatment [Ambient] × [D].

^b^*n* = 3 for treatment [eT × eCO2].

**Table 3:** Individual and combined effects of eT, eCO_2_, and drought in soil parameters, organic and inorganic pools and process rates.

	Treatment effects											
	eT vs. eCO_2_ dataset						Drought dataset					
	[eT]		[eCO_2_]		[eT] × [eCO_2_]		[eTeCO_2_]		[D]		[eT × eCO_2_] × [D]	
	*F*	*p*	*F*	*p*	*F*	*p*	*F*	*p*	*F*	*p*	*F*	*p*
Soil parameters												
SWC	3.140	0.09	0.72	0.40	1.180	0.29	1.740	0.21	1164.59	**2.24** **×** **10**^−**16**^	0.621	0.42
pH	0.557	0.47	0.01	0.93	1.280	0.27	0.890	0.36	0.28	0.60	0.320	0.57
Soil N	0.128	0.73	1.39	0.26	3.140	0.09	0.262	0.62	1.08	0.31	0.422	0.52
MBN	5.620	**0.03**	0.99	0.33	0.769	0.40	6.110	**0.02**	11.30	**3.00** **×** **10**^−**3**^	0.240	0.63
Extractable organic and inorganic pools												
NO_3_^−^	1.570	0.23	0.40	0.53	0.010	0.89	0.990	0.33	1.03	0.32	1.720	0.20
NH_4_^+^	0.510	0.48	0.05	0.82	0.080	0.77	0.030	0.86	93.35	**4.23** **×** **10**^−**8**^	0.670	0.42
DIN	1.230	0.28	0.31	0.58	0.005	0.94	0.700	0.41	15.24	**1.00** **×** **10**^−**3**^	0.100	0.75
DON	3.120	0.09	0.54	0.47	2.260	0.15	0.007	0.93	10.96	**4.00** **×** **10**^−**3**^	1.070	0.31
TDN	0.140	0.71	1.40	0.25	1.250	0.28	0.770	0.39	26.35	**1.00** **×** **10**^−**4**^	0.037	0.84
DOC	5.330	**0.02**	0.01	0.89	NA	NA	1.680	0.21	24.44	**1.00** **×** **10**^−**4**^	0.000	0.99
Gross process rates												
NH_4_^+^ mineralization	0.560	0.46	0.84	0.37	1.550	0.23	8.180	**0.01**	22.81	**2.00** **×** **10**^−**4**^	5.830	**0.03**
NH_4_^+^ immobilization	0.020	0.88	1.19	0.29	3.110	0.10	2.210	0.16	5.60	**0.03**	0.068	0.79
Nitrification	0.030	0.85	0.95	0.34	0.430	0.51	1.320	0.26	15.26	**1.40** **×** **10**^−**3**^	0.002	0.96

Statistical effects were assessed by a 2-way ANOVA type II or a 2-way ANOVA type III whenever a significant interaction term was found. Least square means (lsmeans) multiple comparison tests were ran on interactive effects. Significant effects from the ANOVA are shown in bold. NA depict variables for which nonparametric tests were used and therefore an interactive effect could not be calculated.

*SWC* soil water content, *MBN* microbial biomass nitrogen, *DIN* dissolved inorganic nitrogen, *DON* dissolved organic nitrogen, *TDN* total dissolved nitrogen, *DOC* dissolved organic nitrogen.

## Results

### Soil biogeochemical variables and processes are sensitive to drought but not to other global change factors

Manipulation of CO_2_ concentration and temperature had little or no effect on soil N and C pools, and N processes in the studied grassland (Table 3). No significant statistical effect was found for [eCO_2_], neither alone nor in combination with [eT], for soil N, SWC, pH and gross N process rates. In contrast, only microbial MBN and DOC increased significantly in response to [eT] alone (Table 3).

Drought had a significant effect on most of the measured variables and processes. SWC decreased from 33% in the ambient plots to around 6% in the drought plots (Tables 2 and 3, *F* = 1164.6, *p* = 2.25 × 10^−16^). In addition, drought caused a loss of 24.6 % (in ambient conditions) and 50% (in future climate scenarios) of aboveground plant biomass, which corresponded to a reduction in plant N uptake of 15.8% and 46.6% respectively (data not shown). Furthermore, MBN was significantly higher in both the drought plots (*F* = 11.31, *p* = 0.003) and in [eT × eCO_2_] plots (*F* = 6.11, *p* = 0.025), with no significant interaction term (Table 3).

There was a significant increase of DIN (*F* = 15.24, *p* = 0.001), DON (*F* = 10.96, *p* = 0.004), TDN (*F* = 26.35, *p* = 0.0001), and DOC (*F* = 24.44, *p* = 0.0001) in the drought plots relative to ambient plots, but no significant effect of [eT × eCO_2_] conditions. The response of inorganic N forms to drought was mainly driven by increases in NH_4_^+^ contents under drought (Table 3; *F* = 93.35, *p* = 4.23  × 10^−8^), since NO_3_^−^ levels remained unchanged. Moreover, N mineralization rates increased significantly in response to individual and combined effects of drought and [eT × eCO_2_] (Table 3). Concomitantly, nitrification rates were significantly higher in the drought plots (Tables 2 and 3). These results should however be interpreted with caution due to the potential short-term rewetting effect introduced by the application of a liquid tracer during the isotope pool dilution experiments [66].

### AOB are the most abundant ammonia-oxidizer group and did not decrease with drought

Betaproteobacterial AOB *amoA* gene copy numbers ranged between 1.43 × 10^9^ and 3.79 × 10^9^ copies g^−1^ DW, indicating that they were the most abundant of all ammonia-oxidizing microorganisms in these montane-grassland plots (Fig. 1). Archaeal *amoA* gene copy numbers ranged between 1.21 × 10^8^ and 2.70 × 10^8^ copies g^−1^ DW, and CMX clade B *amoA* gene copy numbers ranged from 9.10 × 10^7^ to 2.50 × 10^8^ copies g^−1^ DW. We observed no significant effect of individual and combined [eT] and [eCO_2_] effects on *amoA* gene abundance (Fig. 1a). We found an interactive effect of [eTeCO_2_] and [D] in both CMX and AOB (Fig. 1b), although post-hoc analyses revealed significant differences between individual treatments for AOB only (Table S3).

Betaproteobacterial AOB *amoA* transcript copy numbers ranged between 1.14 × 10^7^ and 3.62 × 10^7^ copies g^−1^ DW, whereas AOA and CMX clade B had similar *amoA* transcript copies g^−1^ DW (0.27–0.99 × 10^7^ and 0.23–1.46 × 10^7^, respectively) (Fig. 1). We observed no significant individual [eT] and [eCO_2_] effects on *amoA* transcription (Fig. 1a; Table S2). There was an interactive effect in CMX *amoA* transcription (Fig. 1a), although not supported by the post-hoc analysis (Table S3). On the other hand, both AOA and CMX clade B *amoA* transcript copy numbers decreased significantly in response to drought (Fig. 1b). Notably, AOB *amoA* transcription was not affected by drought, but a significant decrease in AOB *amoA* transcript copy numbers was observed in plots affected by [eT × eCO_2_].

### AOA and NOB community structure is significantly affected by drought

The richness of nitrifying communities in all treatments was relatively low. We recovered 12 CMX *amoA* OTUs, 23 AOA *amoA* OTUs, and 15 AOB *amoA* OTUs which were robustly assigned to the corresponding phylogenetic clades after excluding unspecific OTUs (Figs. S2, S3). Unspecific OTUs comprised 0.01%, 0.05%, and 0.3% of all reads for AOA, CMX, and AOB, respectively, (Table S1) and are therefore unlikely to interfere with the qPCR quantifications. In addition to excluding nonspecific AOA OTUs, the coverage of the qPCR primers was checked in silico due to the use of different AOA primer pairs for archaeal *amoA* quantification and sequencing. This resulted on the exclusion of three more AOA OTUs, which comprised 32% of all reads (Table S1). Notably, CMX clade A *amoA* amplification was not detectable in all samples. Furthermore, 40 *nxrB* OTUs also displayed a robust taxonomic classification within the reference tree after excluding nonspecific OTUs (Table S1, Fig. S4).

**Fig. 2:**
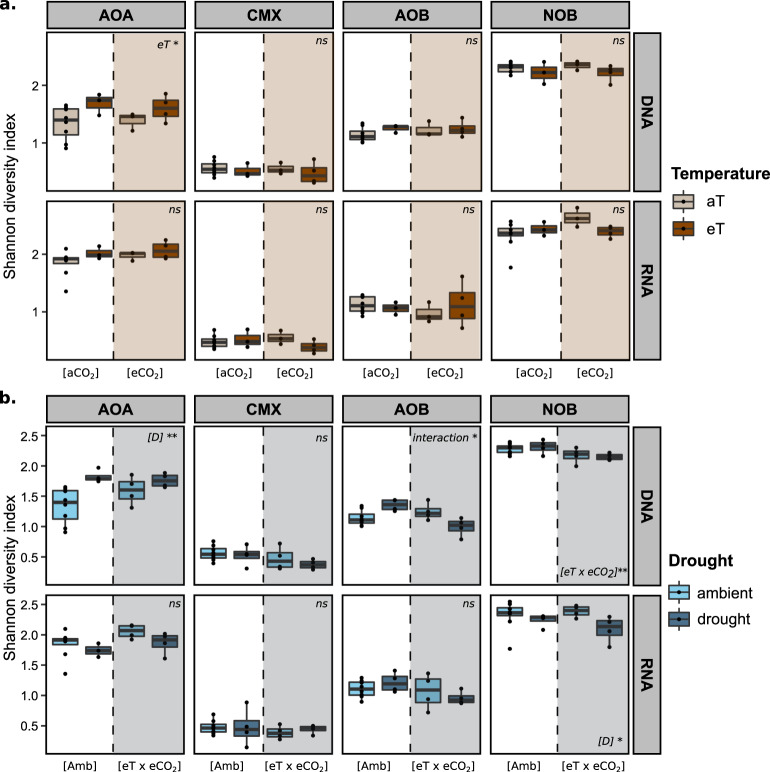
Shannon diversity index based on amoA/nxrB gene and transcript sequences from all microbial groups. **a** Quantifications from plots affected by elevated temperature (eT) and atmospheric CO_2_ concentrations (eCO_2_). The dashed lines represent plots affected by elevated atmospheric CO_2_ conditions (eCO_2_). The colors represent plots affected by either ambient temperature (aT) or elevated temperature (eT). **b** Quantifications from plots affected by future climate (eT x eCO_2_) and drought (D). The dashed lines separate plots affected by drought (D). The colors represent the drought treatment. The caption on the upper right corner of each subplot represents the ANOVA results. *p value <0.05; **p value <0.01. Whenever the ANOVA results showed a significant interaction term, lsmeans multiple comparison tests were ran. For details on the ANOVA and post-hoc tests, see Tables S2 and S3.

**Fig. 3:**
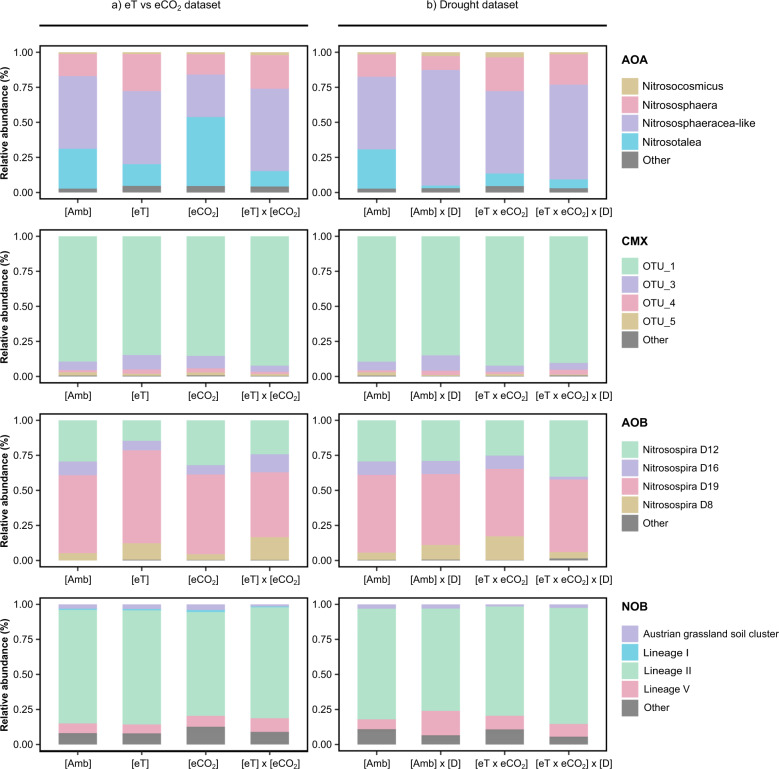
Relative abundance (%) of all nitrifying groups obtained by amoA and nxrB transcript sequences. **a** Data for plots affected by single and interactive effects of elevated temperature (eT) and atmospheric CO_2_ (eCO_2_) concentrations. **b** Plots affected by single and interactive effects of future climate conditions (eT x eCO_2_) and drought (D). The color code represents different genera/OTU/clades/lineages. “Amb” stands for “ambient conditions”. Multiple horizontal lines within the same color represent individual OTUs within a group. Taxa that comprised <0.1% of all reads per treatment are grouped as “Other”.

The most abundant AOA *amoA* gene and transcript OTU was affiliated with genus *Nitrosotalea* and comprised 12–65% of the reads per treatment. The remaining OTUs were affiliated with members of the family *Nitrososphaeraceae*, which comprises the genera *Nitrososcosmicus* and *Nitrososphaera*. Within the *Nitrososphaeraceae*, we observed a sister group to the genus *Nitrososphaera*, comprising ten OTUs and 13–37% of the reads per treatment (Fig. S2a). Statistical analyses showed that the AOA *amoA* gene community structure was significantly affected by [eT] alone (PERMANOVA, *F* = 8.50; *p* = 0.02; Table S4; Fig. S5a), and plots on which temperature was manipulated individually showed a higher Shannon index (ANOVA, *F* = 5.18; *p* = 0.03; Fig. 2a). In addition, variance partitioning revealed a significant difference in AOA *amoA* gene and transcript community structure in response to drought (PERMANOVA, *F* = 36.48, *p* ≤ 0.001; and *F* = 3.91, *p* = 0.04, respectively) (Fig. 3b; Table S4; Fig. S5b). Changes in community structure resulted in a significantly higher Shannon diversity index of AOA *amoA* gene (ANOVA, *F* = 10.908, *p* = 0.004) in the drought plots (Fig. 2b). The changes in AOA community composition at both transcript and gene levels reflected a clear decrease in the relative abundance of *Nitrosotalea*-affiliated OTUs in response to individual manipulations of drought (Fig. 3a; Fig. S5b) and [eT] (Fig. 3a; Fig. S5a).

Regarding CMX clade B *amoA* gene and transcript diversity, a clear dominance of one single OTU, which comprised an average of 87.3% and 87.5% of gene and transcript reads per treatment, respectively, (Fig. 3; Fig. S5), was observed. There were no significant effects of any tested variable on CMX clade B community composition and diversity indexes.

The betaproteobacterial AOB *amoA* diversity was dominated by OTUs affiliated with the genus *Nitrosospira*, and the abundance of *Nitrosomonas*-related *amoA* OTUs was very low (average of 0.24% and 0.73% of *amoA* gene and transcript reads per treatment, respectively). At the transcript level, the most abundant OTU was most similar to an uncultured *Nitrosospira* clade D19 OTU (100% identity), which comprised an average of 53% of the reads per treatment (Fig. 3), dropping to 24.4% at the gene level (Fig. S4). On the other hand, the most abundant OTU at the gene level was most similar to an uncultured *Nitrosospira* clade D12 OTU (100% identity), with an average of 56.7% of the reads per treatment (Fig. S4). The most abundant transcript OTU was 87.6% identical to the most abundant gene OTU. Variance partitioning showed a significant interaction effect of [eT × eCO_2_] × [D] on the diversity and relative abundance of AOB *amoA* transcripts (PERMANOVA, *F* = 5.34, *p* = 0.02; Table S4). Notably, this treatment showed the lowest Shannon diversity index across all treatments (Shannon = 0.95 ± 0.05; Fig. 2b). At the AOB *amoA* gene level, a significant increase in the Shannon index was observed in ambient plots versus [eT × eCO_2_] plots, only under drought conditions (Fig. 2b; Tables S2 and S4).

*Nitrospira nxrB* OTUs were affiliated with lineage 1, 2, 5, and 6, with a clear dominance of lineage 2, which comprised an average of 88.9% and 79.9% of gene and transcripts, respectively (Fig. 3; Fig. S5). The closest culturable representative of the most abundant OTU was *Nitrospira japonica* (99.92% identity), and most of the OTUs clustered together with environmental sequences from Austrian and Namibian soil clusters (Fig. S4). Amplicon sequencing showed that NOB were the most diverse group of nitrifiers, showing the highest Shannon index values of all microbial groups. (Fig. 2). Variance partitioning showed that [eT] alone caused significant differences in *nxrB* gene community structure (PERMANOVA, *F* = 4.06, *p* = 0.02; Table S4; Fig. S5a), although there were no significant differences in alpha diversity. Drought caused significant differences in both *nxrB* gene and transcript community composition (PERMANOVA, *F* = 5.34, *p* = 0.02, PERMANOVA, *F* = 6.53, *p* < 0.01; Table S4). These changes were coupled with significantly lower levels of alpha diversity under drought at the transcript level (ANOVA, *F* = 5.75, *p* = 0.03; Fig. 2b), although the same trend was not observed at the gene level. Contrastingly, *nxrB* gene community structure was significantly affected by [eT × eCO_2_] (PERMANOVA, *F* = 5.52, *p* = 0.02; Table S4), which was reflected in significantly lower Shannon index values (ANOVA, *F* = 9.12, *p* = 0.008; Fig. 2b).

## Discussion

Ammonia-oxidation, usually regarded as the rate-limiting step of nitrification [67], is mediated by specific groups of bacteria and archaea. Therefore, climate change induced alterations in nitrifier community structure could affect soil nitrification to a higher degree than other N cycling processes, such as protein decomposition [11, 68] which are carried out by broader sets of microorganisms. In addition, since the discovery of complete nitrifiers, there is a need for the reassessment of the relative role of each microbial group in ammonia oxidation under climate change. Here we report an in-depth assessment on the effect of multiple climate change drivers on soil nitrification, including (i) the quantification of functional genes and transcripts by quantitative PCR, (ii) a census of the nitrifying microorganisms by sequencing at the functional gene and transcript levels, and (iii) an estimation of gross ammonification and nitrification rates.

In contrast to our first hypothesis we found that the nitrification process was relatively resistant to increases in atmospheric CO_2_ with no significant changes in gross nitrification and in nitrifier functional gene abundance and expression. Effects of increased atmospheric CO_2_ on soil microbial processes are primarily determined by changes in plant belowground carbon inputs and in plant nutrient uptake [69], where rhizodeposition can promote heterotrophic microbial activity, but greater plant N uptake may cause substrate (NH_4_^+^) limitation for nitrifiers. At our site, no increase in above and belowground net primary productivity was found in response to elevated CO_2_ or temperature (Canarini et al., in preparation). Likewise, eCO_2_ did not affect gross N mineralization and NH_4_^+^ levels, highlighting that substrate availability for nitrifiers did not change in response to eCO_2_. In addition, other studies also reported very few effects of elevated atmospheric CO_2_ concentration on belowground N processes in heathlands and temperate grasslands [70–72] showing that soil parameters such as pH and inorganic N concentrations likely play a more significant role than increased atmospheric CO_2_ on overall nitrification.

Elevated temperature alone also did not significantly affect most of the soil processes and variables studied. More importantly, in contrast to our second hypothesis, elevated temperature did not affect the abundance, composition, and activity of soil nitrifier communities, indicating a high tolerance of the nitrification process to temperature in this ecosystem. Also, our results are in line with meta-analyses that observed that the effect of temperature on N cycling processes is less pronounced in grasslands, in comparison with other ecosystem types [36, 73]. In addition, other studies in grassland systems also reported no significant effects of elevated temperature on nitrification [72, 74]. Our results indicate that increased temperature might have altered abiotic and biotic soil parameters that may be masking individual temperature effects. Specifically, the soil water content at our field site was found to decrease throughout summer (Simon et al., under review) which can constrain substrate availability and/or accessibility to nitrifiers and mask potential stimulatory effect of warming on the nitrification process. Also, plant production of biological nitrification inhibitors (BNIs) could explain these results. However, BNIs were not assessed in this study, and their importance has mostly been reported in N-limited ecosystems [75, 76].

We further hypothesized that drought would reduce the abundance and activity of all nitrifying groups but found that responses to drought were group specific. The *amoA* expression levels of AOA and CMX clade B significantly decreased, whereas AOB either maintained (eT × eCO_2_ plots) or increased (under ambient temperature and CO_2_) their *amoA* transcription levels under drought. Drought can directly affect microbial growth by reducing the soil water potential and forcing microorganisms to invest into osmoregulation rather than growth and replication [33]. It can also have indirect effects on microbial communities by reducing plant belowground C allocation [77, 78], and by changing the availability and mobility of nutrients through a reduced soil pore connectivity and reduced plant uptake [79]. Studies have shown that plants can alter their root exudate abundance and composition during drought periods [80] as an attempt to better cope with the osmotic stress and/or to recruit particular fungi to the vicinity of the roots [81, 82]. Within root exudates, plants can also actively secrete BNIs, but given that fertilized grasslands are often not N limited, and our results point to higher NH_4_^+^ levels under drought, there is little evidence to support a relevant role for BNIs in these systems [76, 83]. Also, at the same study site, a decrease in plant biomass has been observed under drought, as well as a reduction in total plant N uptake. As a consequence of the plant response to drought, organic and inorganic dissolved N forms accumulated in the soil (Table 2) as reported in other studies [33]. The same pattern was observed with regards to microbial biomass N. Previous studies reported an increase in microbial biomass with drought, and have indicated that major pools of C- and N-based microbial metabolites are dynamic in response to drought [84, 85]. The accumulation of organic and inorganic N forms might have created optimal conditions for the proliferation of AOM with low ammonia (NH_3_) affinities and high V_max_, such as AOB, as opposed to AOA and CMX [12]. AOA have been reported to be more sensitive to perturbations and variations in NH_4_^+^ concentrations when compared with AOB [11, 86]. Also, the nitrification activity of both AOA and CMX saturates faster due to lower maximum rates of NH_3_ oxidation, compared with AOB [12]. However, no pure culture representative exists for CMX clade B yet, and the kinetic parameters of *Nitrospira inopinata*—the only CMX *Nitrospira* clade A isolate so far—might not be representative of all CMX members [12]. AOB, and particularly members of the genus *Nitrosospira*, are ubiquitous in grasslands and tolerate high concentrations of NH_4_^+^ [87]. In addition, genes involved in the protection of bacterial cells from hypoosmotic stress have been found in *N. multiformis*, showing that these organisms are well adapted to changes in soil water potential. However, osmoprotectants have also been detected in genomes of cultivated AOA [88–91], indicating that these organisms can also adapt to osmotic stress. Nevertheless, a dominance of AOB in managed grasslands has been reported due to frequent fertilization events [13, 14], and AOB are known to outcompete AOA in substrate-rich environments [9].

Along with an overall decrease in archaeal *amoA* gene and transcript copy number in response to drought, we found a reduction in the relative abundance of AOA OTUs affiliated to *Nitrosotalea*. This could be explained by the accumulation of organic compounds under drought, some of which were shown to inhibit *Nitrosotalea* activity and growth in pure cultures [92] compared with other AOA such as *N. viennensis* and *N. gargensis* [88, 93]. Furthermore, inhibition by organic compounds has been shown to be stronger in AOA than in AOB, although it varies between AOA strains [94, 95].

Even though we lack quantitative data on *nxrB* gene and transcript abundance due to still unresolved methodological challenges, we observed that the existing *Nitrospira-like* NOB community structure differed significantly in the drought plots compared with the non-drought plots, which was reflected on a decrease in the Shannon diversity index at the transcript level. *Nitrospira* were shown to have a high affinity for nitrite, and often dominate NOB communities in N-limited soils [96]. Given that NO_2_^−^ often does not accumulate in soils, *Nitrospira* are considered to be the main nitrite-oxidizing genus in these ecosystems [97, 98]. Regardless of the decrease in diversity under drought, *Nitrospira* sublineage II remained the most abundant sublineage. Pure culture studies have reported a high metabolic flexibility of members of this sublineage, and it is possible that some microorganisms affiliated with sublineage II are better able to cope with the higher osmotic stress and levels of inorganic N imposed by drought [99, 100]. In addition, another study has reported higher rates of autotrophic growth of a *Nitrospira* sublineage II phylotype upon the addition of NH_4_^+^ [101].

Emission of the greenhouse gas N_2_O has been shown to increase up to eightfold following the end of a drought period in grassland ecosystems, and a single short rain event can increase the annual net N_2_O flux between 2 and 50% [102]. Therefore, our findings may also have broader implications since a drought-induced alteration in nitrifier community structure in favor of AOB over AOA and CMX might lead to higher N_2_O emissions upon rewetting. In soil, N_2_O is produced by denitrifying and ammonia-oxidizing microorganisms [103]. Within the latter group, AOB produce relatively high yields of N_2_O via hydroxylamine oxidation under oxic conditions [104], while AOA and CMX produce much lower yields of N_2_O during aerobic ammonium oxidation [105, 106]. Changes in redox potential could affect nitrification processes, specifically at the interface between aerobic and (partially) anaerobic soil portions. However, the soils in this study did not reach anaerobic conditions (maximum 34% soil water content), therefore making it unlikely that redox changes would have played a significant role.

Finally, we found few to no significant interactive effects between the different climate change drivers. Global reviews have shown that the interaction of different climate change factors can lead to nonadditive effects [6], although a more recent meta-analysis showed that antagonistic and synergistic effects are quite rare [7]. A recent study in a grassland system assessed the effect of elevated temperature and elevated CO_2_ and found that while combinatory effects were antagonistic, they were mostly nonsignificant [72]. Regarding nitrifier community composition, only active AOB were significantly affected by the interactive effect of future climate conditions and drought [eTeCO_2_ × [D]). Under these conditions, the AOB diversity and abundance were lowest, as assessed by *amoA* amplicon sequencing and gene and transcript copy number quantification, which agrees with our last hypothesis.

In conclusion, in our study we demonstrated that nitrifying communities in grassland soils were remarkably unresponsive to elevated CO_2_ and elevated temperature, alone or in combination, resulting in unaltered nitrification rates. We also showed that drought significantly changed the structure of the existing nitrifying communities, possibly through a strong reduction of plant biomass and N uptake. We hypothesize that the specific conditions created by drought (such as the accumulation of NH_4_^+^ and organic N, and, reduced pore connectivity) resulted in favorable niches for AOB, that showed higher or unaltered levels of *amoA* transcription and drove the observed peak in nitrification rates at the expense of ammonium-oxidizing archaea and comammox *Nitrospira*. Nevertheless, caution should be taken when interpreting nitrification rates due to the potential short-term rewetting effect introduced by the application of the liquid tracer (see Methods section). Future climate conditions further interacted with drought and caused an antagonistic effect on the diversity and abundance of this group, without reducing gross nitrification rates. Given that a shift in nitrifier community structure towards AOB could potentially result in higher N_2_O emission rates at the end of the drought period, special emphasis should be put into understanding the sensitivity of different nitrifiers to individual and combined global change variables in terrestrial ecosystems.

## Supplementary information

Supplementary Materials and Methods

Table S1

Table S2

Table S3

Table S4

Figure S1

Figure S2

Figure S3

Figure S4

Figure S5

Supplementary Data
