# Supplementary material for: Composition and activity of nitrifier communities in soil are unresponsive to elevated temperature and CO2, but strongly affected by drought
Source: ISME J. 2020 Aug 7;14(12):3038–53. doi: 10.1038/s41396-020-00735-7 (PMC7784676; doi:10.1038/s41396-020-00735-7)
Supplement: Supplementary file 1 — Supplementary Materials and Methods [file 41396_2020_735_MOESM1_ESM.docx]

**Supplementary Material and Methods**

***amoA*/*nxrB* gene and transcript amplification conditions**

The PCR reactions were performed with a Biomek NXP span 8 automated liquid handler (Beckman-Coulter). First-step PCR amplifications were performed in triplicates of 25 µl reaction volume, containing: 1x DreamTaq Green Buffer, 0.2 mmol l^-1^ dNTPs, 0.5 to 1 µg µl^-1^ BSA, 1.25 to 2.5 U ml^-1^ DreamTaq Green DNA Polymerase (Thermo Fisher Scientific) and 0.5 µmol l^-1^ of each head primer **(Table 1)**. The amount of template ranged between 5 and 15 ng DNA, with an average input of 9.8 ng DNA. Amplification of all functional genes and transcripts was performed with 5 min initial denaturation at 94 °C, followed by 35 cycles of 30 s denaturation at 94 °C, 45 s primer annealing at the corresponding temperature **(Table 1)**, and 10 min of product elongation at 72 °C. For each gene, a positive control and a negative control were included. The negative control included each set of primer pairs and no template, whereas the positive control included each set of primer pairs and the corresponding qPCR standard. For NOB, a pure culture of *Nitrospira moscoviensis* was used as positive control.

Following the first step PCR amplification, samples were purified and normalized using the SequalPrep Clean-Up and Normalization kit (Invitrogen) and 9 µl of the normalized sample was used for the barcoding PCR reaction, containing: 1x DreamTaq Green Buffer, 0.2 mmol l^-1^ dNTPs, 0.08 µg µl^-1^ BSA, 1.25 U ml^-1^ DreamTaq Green DNA Polymerase (Thermo Fisher Scientific), and 0.8 µmol l^-1^ of a head-barcode primer **(Supplementary Datafile 1).** The following program was used for the barcoding PCR: 94 °C for 5 min followed by 6 cycles of 94 °C for 30 s, 52 °C for 45 s and 72 °C for 45 s, and a single step of final elongation at 72 °C for 10 min. Following the barcoding PCR step, samples were again cleaned and normalized with the SequalPrep Kit and pooled in equimolar amounts.

|  |  |  |  |  |  |  |
| --- | --- | --- | --- | --- | --- | --- |

**Table 1.** Primer pairs and amplification conditions used in this study.

| Primer pair | Target organism | Amplicon size (nt) | BSA concentration (µg/µl^-1^) | Annealing temperature (℃) (PCR/qPCR) | Reference |
| --- | --- | --- | --- | --- | --- |
| comB-244F/comB-659R^1^ | CMX | 415 | 0.5 | 57/56 | (1) |
| amoA1F/amoA2R^1^ | AOB | 491 | 1 | 60/58 | (2) |
| 104F/616R^1^ | AOA | 512 | 0.5 | 55 | (3, 4) |
| GenAOAF/GenAOAR^2^ | AOA | 200 | 0.5 | 55 | (5) |
| 169F/638R^1^ | NOB *Nitrospira* | 485 | 0.5 | 57 | (6) |

^1^ primer pairs used for sequencing had a linker sequence (GCTATGCGCGAGCTGC) added at the 5' end

^2^ primer pair used for AOA qPCR

***amoA* gene and transcript quantification by qPCR**

All qPCR assays were performed on a Bio-Rad C1000 CFX96 Real-Time PCR system (Bio-Rad, Hercules, CA, USA) with Bio-Rad iQ SYBR Green Supermix (Bio-Rad, Hercules, CA, USA), containing 50 U ml^-1^ iTaq DNA polymerase, 0.4 mmol l^-1^ dNTPs, 100 mmol l^-1^ KCl, 40 mmol l^-1^ Tris-HCl, 6 mmol l^-1^ MgCl_2_, 20 mmol l^-1^ fluorescein, and stabilizers. The respective primers were added to a final concentration of 0.5 µmol l^-1^, and BSA (Thermo Fisher Scientific) was added at concentrations of 0-1 µg µl^-1^ (**Table 1**). DNA and cDNA (2-4 µl) were added to each PCR reaction to a final volume of 20 μl reaction mix. Amplification of all *amoA* gene and transcript copy numbers was performed with 5 min initial denaturation at 95 °C, followed by 45 cycles of 30 s denaturation at 95 °C, 45 s primer annealing at the corresponding temperature **Table 1)**, and 1 min of product elongation at 72 °C. Fluorescence intensity was recorded at 72 °C for amplicon quantification. After amplification, an amplicon melting curve was recorded in 0.5 °C steps at 40-96 °C to confirm product specificity.

For AOA *amoA* gene amplification, we evaluated several published primer pairs, with the goal of identifying a primer pair suitable for qPCR assays and amplicon sequencing in our samples. The primer pair used for amplicon sequencing gives good phylogenetic resolution due to the long amplicon size (4, 6). It did however significantly decrease qPCR efficiency. Therefore, we used another AOA *amoA* gene-targeted primer pair with sufficient specificity and efficiency for qPCR assays and with similar taxonomic coverage (5). We additionally checked the coverage of the qPCR primers on the retrieved OTUs.

**References**

1. Pjevac P, Schauberger C, Poghosyan L, Herbold CW, van Kessel MAHJ, Daebeler A, Steinberger M, Jetten MSM, Lücker S, Wagner M, Daims H. 2017. *AmoA*-targeted polymerase chain reaction primers for the specific detection and quantification of comammox *Nitrospira* in the environment. Front Microbiol 8:1–11.

2. Rotthauwe JH, Witzel KP, Liesack W. 1997. The ammonia monooxygenase structural gene *amoA* as a functional marker: molecular fine-scale analysis of natural ammonia-oxidizing populations. Appl Environ Microbiol 63:4704 LP – 4712.

3. Alves RJE, Wanek W, Zappe A, Richter A, Svenning MM, Schleper C, Urich T. 2013. Nitrification rates in Arctic soils are associated with functionally distinct populations of ammonia-oxidizing archaea. ISME J 7:1620–31.

4. Tourna M, Freitag TE, Nicol GW, Prosser JI. 2008. Growth, activity and temperature responses of ammonia-oxidizing archaea and bacteria in soil microcosms. Environ Microbiol 10:1357–1364.

5. Meinhardt KA, Bertagnolli A, Pannu MW, Strand SE, Brown SL, Stahl DA. 2015. Evaluation of revised polymerase chain reaction primers for more inclusive quantification of ammonia-oxidizing archaea and bacteria. Environ Microbiol Rep 7:354–363.

6. Pester M, Maixner F, Berry D, Rattei T, Koch H, Lücker S, Nowka B, Richter A, Spieck E, Lebedeva E, Loy A, Wagner M, Daims H. 2014. *NxrB* encoding the beta subunit of nitrite oxidoreductase as functional and phylogenetic marker for nitrite-oxidizing *Nitrospira*. Environ Microbiol 16:3055–3071.
