# Supplementary material for: Composition and activity of nitrifier communities in soil are unresponsive to elevated temperature and CO2, but strongly affected by drought
Source: ISME J. 2020 Aug 7;14(12):3038–53. doi: 10.1038/s41396-020-00735-7 (PMC7784676; doi:10.1038/s41396-020-00735-7)
Supplement: Supplementary file 2 — Table S1 [file 41396_2020_735_MOESM2_ESM.docx]

| Gene | Reads retrieved^1^ | Clustering cut-off (%) | OTUs retrieved | Unspecific OTUs | Read number unspecific OTUs | Unspecific OTUs (% in dataset) | Unspecific OTUs (maximum read number per sample) |
| --- | --- | --- | --- | --- | --- | --- | --- |
|  |  |  |  |  |  |  |  |
| *amoA* (AOB) | 57847 | 95 | 39 | 24 | 228 | 0.39 | 25 |
| *amoA* (AOA)^2^ | 157030 | 96 | 29 | 3 (3) | 23 (49517) | 0.01 (32) | 6 (2158) |
| *amoA* (CMX) | 247661 | 95 | 19 | 7 | 131 | 0.05 | 30 |
| *nxrB* (NOB) | 66900 | 95 | 141 | 101 | 3081 | 4.61 | 335 |
|  |  |  |  |  |  |  |  |
| ^1^ read number after removal of positive and negative sequencing controls | | | | | |  |  |
| ^2^ sequencing of one sample from the transcript dataset [Amb] x [D] failed | | | | | |  |  |

**Table S1**. Amplicon data summary statistics derived from *amoA*/*nxrB* gene and transcript sequences. Numbers. Numbers in parenthesis indicate values after excluding all AOA OTUs not covered by the qPCR primers.
