# Supplementary material for: Composition and activity of nitrifier communities in soil are unresponsive to elevated temperature and CO2, but strongly affected by drought
Source: ISME J. 2020 Aug 7;14(12):3038–53. doi: 10.1038/s41396-020-00735-7 (PMC7784676; doi:10.1038/s41396-020-00735-7)
Supplement: Supplementary file 3 — Table S2 [file 41396_2020_735_MOESM3_ESM.docx]

**Table S2.** Individual and combined effects of eT, eCO_2_ and drought on the Shannon index based on *amoA* and *nxrB* gene and transcript sequences, as well as on the *amoA* gene and transcript qPCR quantification. Main findings are shown in Fig.1 and Fig.2 Statistical effects were assessed by a 2-way ANOVA type II or a 2-way ANOVA type III whenever a significant interaction term was found. Least square means (lsmeans) post-hoc tests were ran on interactive effects. Significant effects from the ANOVA are shown in bold. NA depict variables for which non-parametric tests were used and therefore an interactive effect could not be calculated.

|  |  | ***Treatment effects*** | | | |  |  |  |  |  |  |  |  |
| --- | --- | --- | --- | --- | --- | --- | --- | --- | --- | --- | --- | --- | --- |
|  |  |  |  |  |  |  |  |  |  |  |  |  |  |
|  |  | *eT vs eCO_2_ experiment* | | | | | | *Drought experiment* | | | | | |
|  |  | [eT] | | [eCO_2_] | | [eT] x [eCO_2_] | | [eTeCO_2_] | | [D] | | [eT x eCO_2_] x [D] | |
|  |  | F | *p* | F | *p* | F | *p* | F | *p* | F | p | F | *p* |
| Gene | ***Shannon index*** |  |  |  |  |  |  |  |  |  |  |  |  |
|  | AOA *amoA* | 5.189 | **0.039** | 0.007 | 0.933 | 0.282 | 0.604 | 1.269 | 0.277 | 10.908 | **0.004** | 2.201 | 0.157 |
|  | CMX *amoA* | 0.802 | 0.386 | 0.122 | 0.732 | 0.058 | 0.814 | 0.620 | 0.444 | 0.102 | 0.754 | 2.745 | 0.120 |
|  | AOB *amoA* | 1.204 | 0.291 | 0.490 | 0.495 | 0.499 | 0.491 | 4.593 | **0.048** | 0.104 | 0.752 | 14.812 | **0.001** |
|  | NOB *nxrB* | 3.189 | 0.096 | 0.127 | 0.727 | 0.149 | 0.705 | 9.108 | **0.008** | 0.033 | 0.857 | 0.348 | 0.564 |
|  | ***qPCR quantification*** |  |  |  |  |  |  |  |  |  |  |  |  |
|  | AOA *amoA* | 0.734 | 0.406 | 0.474 | 0.505 | 0.650 | 0.434 | 0.767 | 0.394 | 1.636 | 0.219 | 0.929 | 0.350 |
|  | CMX *amoA* | 2.435 | 0.140 | 0.001 | 0.974 | 4.304 | 0.056 | 1.036 | 0.324 | 0.001 | 0.973 | 6.768 | **0.019*** |
|  | AOB *amoA* | 0.173 | 0.684 | 0.028 | 0.868 | 0.760 | 0.398 | 10.336 | **0.005** | 2.255 | 0.153 | 6.881 | **0.018*** |
| Transcript | ***Shannon index*** |  |  |  |  |  |  |  |  |  |  |  |  |
|  | AOA *amoA* | 2.380 | 0.145 | 1.108 | 0.310 | 0.178 | 0.679 | 3.007 | 0.083 | 2.064 | 0.151 | NA | NA |
|  | CMX *amoA* | 0.620 | 0.444 | 0.102 | 0.754 | 2.745 | 0.120 | 0.745 | 0.401 | 0.090 | 0.769 | 0.127 | 0.727 |
|  | AOB *amoA* | 0.002 | 0.964 | 0.576 | 0.460 | 0.022 | 0.884 | 3.471 | 0.081 | 0.250 | 0.624 | 0.265 | 0.614 |
|  | NOB *nxrB* | 0.788 | 0.390 | 3.541 | 0.081 | 4.574 | 0.051 | 0.061 | 0.808 | 5.924 | **0.027** | 0.800 | 0.384 |
|  | ***qPCR quantification*** |  |  |  |  |  |  |  |  |  |  |  |  |
|  | AOA *amoA* | 0.079 | 0.783 | 0.856 | 0.371 | 0.136 | 0.718 | 0.086 | 0.773 | 5.554 | **0.032** | 0.618 | 0.443 |
|  | CMX *amoA* | 1.271 | 0.278 | 0.017 | 0.897 | 5.672 | **0.031*** | 1.060 | 0.318 | 9.039 | **0.008** | 0.701 | 0.415 |
|  | AOB *amoA* | 0.324 | 0.578 | 1.233 | 0.286 | 2.145 | 0.165 | 9.115 | **0.008** | 1.468 | 0.243 | 2.212 | 0.156 |
|  |  |  |  |  |  |  |  |  |  |  |  |  |  |
| ^*^ lsmeans multiple comparison tests were run on significant interactive effects (Table S3) | | | | | | | | | | | | | |
