# Supplementary material for: Composition and activity of nitrifier communities in soil are unresponsive to elevated temperature and CO2, but strongly affected by drought
Source: ISME J. 2020 Aug 7;14(12):3038–53. doi: 10.1038/s41396-020-00735-7 (PMC7784676; doi:10.1038/s41396-020-00735-7)
Supplement: Supplementary file 4 — Table S3 [file 41396_2020_735_MOESM4_ESM.docx]

**Table S3.** Results from the post-hoc tests (Least square means multiple comparison tests) ran only when a significant ANOVA type III interaction effect was observed on the Shannon Index calculated from functional gene and transcript sequencing data, functional gene/transcript quantification by qPCR and on NH_4_^+^ mineralization rates. Groups sharing a letter are not significantly different (Tukey-adjusted comparisons).

| **Dataset** | **Variable** | **Treatment** | **Treatment** | **lsmean** | **SE** | **df** | **lower CL** | **upper CL** | **group** |
| --- | --- | --- | --- | --- | --- | --- | --- | --- | --- |
| Drought | AOB *amoA* gene Shannon Index | [eT x eCO_2_] | drought | 0.99 | 0.07 | 16 | 0.80 | 1.17 | a |
|  |  | ambient | ambient | 1.14 | 0.05 | 16 | 1.01 | 1.27 | ab |
|  |  | [eT x eCO_2_] | ambient | 1.24 | 0.07 | 16 | 1.06 | 1.42 | ab |
|  |  | ambient | drought | 1.35 | 0.07 | 16 | 1.17 | 1.53 | b |
|  | AOB *amoA* gene qPCR quantification | [eT x eCO_2_] | drought | 1.42E+09 | 4.34E+08 | 16 | 2.07E+08 | 2.64E+09 | a |
|  |  | ambient | ambient | 2.12E+09 | 3.07E+08 | 16 | 1.26E+09 | 2.98E+09 | a |
|  |  | [eT x eCO_2_] | ambient | 1.88E+09 | 4.34E+08 | 16 | 6.63E+08 | 3.10E+09 | a |
|  |  | ambient | drought | 3.80E+09 | 4.34E+08 | 16 | 2.58E+09 | 5.02E+09 | b |
|  | CMX *amoA* gene qPCR quantification | [eT x eCO_2_] | drought | 9.10E+07 | 2.85E+07 | 16 | 1.12E+07 | 1.71E+08 | a |
|  |  | ambient | ambient | 1.19E+08 | 2.01E+07 | 16 | 6.26E+07 | 1.75E+08 | a |
|  |  | [eT x eCO_2_] | ambient | 1.61E+08 | 2.85E+07 | 16 | 8.14E+07 | 2.41E+08 | a |
|  |  | ambient | drought | 1.87E+08 | 2.85E+07 | 16 | 1.08E+08 | 2.67E+08 | a |
|  | NH_4_^+^ mineralization | [eT x eCO_2_] | drought | 6.97 | 0.568 | 15 | 5.36 | 8.57 | b |
|  |  | ambient | ambient | 2.72 | 0.402 | 15 | 1.58 | 3.86 | a |
|  |  | [eT x eCO_2_] | ambient | 2.97 | 0.568 | 15 | 1.36 | 4.57 | a |
|  |  | ambient | drought | 4.03 | 0.656 | 15 | 2.18 | 5.89 | a |
| eT vs eCO_2_ | CMX *amoA* transcript gene qPCR quantification | ambient | ambient | 6.19E+06 | 1.67E+06 | 14 | 1.43E+06 | 1.09E+07 | a |
|  |  | + 3 ºC | + 300 ppm | 9.22E+06 | 2.36E+06 | 14 | 2.49E+06 | 1.59E+07 | a |
|  |  | ambient | + 300 ppm | 1.22E+07 | 2.72E+06 | 14 | 4.46E+06 | 2.00E+07 | a |
|  |  | + 3 ºC | ambient | 1.46E+07 | 2.72E+06 | 14 | 6.86E+06 | 2.24E+07 | a |
