# Supplementary material for: Composition and activity of nitrifier communities in soil are unresponsive to elevated temperature and CO2, but strongly affected by drought
Source: ISME J. 2020 Aug 7;14(12):3038–53. doi: 10.1038/s41396-020-00735-7 (PMC7784676; doi:10.1038/s41396-020-00735-7)
Supplement: Supplementary file 5 — Table S4 [file 41396_2020_735_MOESM5_ESM.docx]

| **Microbial group** | **Gene/transcript** | **Dataset** | **Factors** | **Df** | **SumsOfSqs** | **MeanSqs** | **F Model** | **R^2^** | ***p*-value** |
| --- | --- | --- | --- | --- | --- | --- | --- | --- | --- |
| AOA | Transcript | eT vs eCO_2_ | [eT] | 1 | 0.0198 | 0.0198 | 2.3727 | 0.1129 | 0.1712 |
|  |  |  | [eCO_2_] | 1 | 0.0253 | 0.0253 | 3.0277 | 0.1441 | 0.1095 |
|  |  |  | [eT] x [eCO_2_] | 1 | 0.0134 | 0.0134 | 1.6080 | 0.0765 | 0.2617 |
|  |  |  | Residuals | 14 | 0.1169 | 0.0083 |  | 0.6664 |  |
|  |  |  | Total | 17 | 0.1754 |  |  | 1.0000 |  |
|  |  | Drought | [eTeCO_2_] | 1 | 0.0102 | 0.0102 | 1.7349 | 0.0765 | 0.2603 |
|  |  |  | [D] | 1 | 0.0298 | 0.0298 | 5.0414 | 0.2223 | **0.0410** |
|  |  |  | [eT x eCO_2_] x [D] | 1 | 0.0053 | 0.0053 | 0.8991 | 0.0397 | 0.4432 |
|  |  |  | Residuals | 15 | 0.0886 | 0.0059 |  | 0.6615 |  |
|  |  |  | Total | 18 | 0.1339 |  |  | 1.0000 |  |
|  | Gene | eT vs eCO_2_ | [eT] | 1 | 0.0371 | 0.0371 | 8.5024 | 0.3669 | **0.0270** |
|  |  |  | [eCO_2_] | 1 | 0.0035 | 0.0035 | 0.8081 | 0.0349 | 0.3946 |
|  |  |  | [eT] x [eCO_2_] | 1 | -0.0006 | -0.0006 | -0.1362 | -0.0059 | 0.8191 |
|  |  |  | Residuals | 14 | 0.0611 | 0.0044 |  | 0.6041 |  |
|  |  |  | Total | 17 | 0.1011 |  |  | 1.0000 |  |
|  |  | Drought | [eTeCO_2_] | 1 | 0.0466 | 0.0466 | 15.9080 | 0.2398 | **0.0085** |
|  |  |  | [D] | 1 | 0.1068 | 0.1068 | 36.4800 | 0.5500 | **0.0003** |
|  |  |  | [eT x eCO_2_] x [D] | 1 | -0.0060 | -0.0060 | -2.0600 | -0.0311 | 0.9924 |
|  |  |  | Residuals | 16 | 0.0469 | 0.0029 |  | 0.2412 |  |
|  |  |  | Total | 19 | 0.1943 |  |  | 1.0000 |  |

**Table S4.** PERMANOVA results performed on *amoA/nxrB* gene and transcript sequencing data. Statistical significance (*p* < 0.05) is shown in bold.

| **Microbial group** | **Gene/transcript** | **Dataset** | **Factors** | **Df** | **SumsOfSqs** | **MeanSqs** | **F Model** | **R^2^** | ***p*-value** |
| --- | --- | --- | --- | --- | --- | --- | --- | --- | --- |
| CMX | Transcript | eT vs eCO_2_ | [eT] | 1 | 0.0001 | 0.0001 | 0.7914 | 0.0434 | 0.4735 |
|  |  |  | [eCO_2_] | 1 | 0.0000 | 0.0000 | 0.1177 | 0.0065 | 0.6920 |
|  |  |  | [eT] x [eCO_2_] | 1 | 0.0005 | 0.0005 | 3.3477 | 0.1834 | 0.1192 |
|  |  |  | Residuals | 14 | 0.0022 | 0.0002 |  | 0.7668 |  |
|  |  |  | Total | 17 | 0.0028 |  |  | 1.0000 |  |
|  |  | Drought | [eTeCO_2_] | 1 | 0.0000 | 0.0000 | -0.0705 | -0.0048 | 0.5940 |
|  |  |  | [D] | 1 | 0.0001 | 0.0001 | 0.2214 | 0.0151 | 0.5400 |
|  |  |  | [eT x eCO_2_] x [D] | 1 | -0.0010 | -0.0010 | -1.4893 | -0.1016 | 0.9073 |
|  |  |  | Residuals | 16 | 0.0102 | 0.0006 |  | 1.0913 |  |
|  |  |  | Total | 19 | 0.0094 |  |  | 1.0000 |  |
|  | Gene | eT vs eCO_2_ | [eT] | 1 | 0.0003 | 0.0003 | 1.6825 | 0.1088 | 0.2827 |
|  |  |  | [eCO_2_] | 1 | 0.0001 | 0.0001 | 0.5195 | 0.0336 | 0.5116 |
|  |  |  | [eT] x [eCO_2_] | 1 | -0.0001 | -0.0001 | -0.7408 | -0.0479 | 0.8853 |
|  |  |  | Residuals | 14 | 0.0023 | 0.0002 |  | 0.9055 |  |
|  |  |  | Total | 17 | 0.0026 |  |  | 1.0000 |  |
|  |  | Drought | [eTeCO_2_] | 1 | 0.0010 | 0.0010 | 5.6961 | 0.2345 | 0.0652 |
|  |  |  | [D] | 1 | 0.0004 | 0.0004 | 1.9660 | 0.0809 | 0.2448 |
|  |  |  | [eT x eCO_2_] x [D] | 1 | 0.0001 | 0.0001 | 0.6280 | 0.0259 | 0.4697 |
|  |  |  | Residuals | 16 | 0.0029 | 0.0002 |  | 0.6587 |  |
|  |  |  | Total | 19 | 0.0044 |  |  | 1.0000 |  |

| **Microbial group** | **Gene/transcript** | **Dataset** | **Factors** | **Df** | **SumsOfSqs** | **MeanSqs** | **F Model** | **R^2^** | ***p*-value** |
| --- | --- | --- | --- | --- | --- | --- | --- | --- | --- |
| AOB | Transcript | eT vs eCO_2_ | [eT] | 1 | 0.0154 | 0.0154 | 2.5010 | 0.1579 | 0.1673 |
|  |  |  | [eCO_2_] | 1 | -0.0032 | -0.0032 | -0.5126 | -0.0324 | 0.9280 |
|  |  |  | [eT] x [eCO_2_] | 1 | -0.0009 | -0.0009 | -0.1480 | -0.0093 | 0.8175 |
|  |  |  | Residuals | 14 | 0.0863 | 0.0062 |  | 0.8838 |  |
|  |  |  | Total | 17 | 0.0977 |  |  | 1.0000 |  |
|  |  | Drought | [eTeCO_2_] | 1 | -0.0015 | -0.0015 | -0.3808 | -0.0180 | 0.8173 |
|  |  |  | [D] | 1 | 0.0007 | 0.0007 | 0.1652 | 0.0078 | 0.6669 |
|  |  |  | [eT x eCO_2_] x [D] | 1 | 0.0215 | 0.0215 | 5.3407 | 0.2528 | **0.0274** |
|  |  |  | Residuals | 16 | 0.0643 | 0.0040 |  | 0.7574 |  |
|  |  |  | Total | 19 | 0.0849 |  |  | 1.0000 |  |
|  | Gene | eT vs eCO_2_ | [eT] | 1 | 0.0016 | 0.0016 | 1.1925 | 0.0852 | 0.3506 |
|  |  |  | [eCO_2_] | 1 | -0.0004 | -0.0004 | -0.3077 | -0.0220 | 0.8023 |
|  |  |  | [eT] x [eCO_2_] | 1 | -0.0012 | -0.0012 | -0.8916 | -0.0637 | 0.9460 |
|  |  |  | Residuals | 14 | 0.0185 | 0.0013 |  | 1.0005 |  |
|  |  |  | Total | 17 | 0.0185 |  |  | 1.0000 |  |
|  |  | Drought | [eTeCO_2_] | 1 | 0.0030 | 0.0030 | 2.9074 | 0.1339 | 0.1518 |
|  |  |  | [D] | 1 | -0.0013 | -0.0013 | -1.2605 | -0.0580 | 0.9719 |
|  |  |  | [eT x eCO_2_] x [D] | 1 | 0.0041 | 0.0041 | 4.0736 | 0.1875 | 0.0907 |
|  |  |  | Residuals | 16 | 0.0163 | 0.0010 |  | 0.7366 |  |
|  |  |  | Total | 19 | 0.0221 |  |  | 1.0000 |  |

| **Microbial group** | **Gene/transcript** | **Dataset** | **Factors** | **Df** | **SumsOfSqs** | **MeanSqs** | **F Model** | **R^2^** | ***p*-value** |
| --- | --- | --- | --- | --- | --- | --- | --- | --- | --- |
| NOB | Transcript | eT vs eCO_2_ | [eT] | 1 | -0.0001 | -0.0001 | -0.0250 | -0.0015 | 0.8620 |
|  |  |  | [eCO_2_] | 1 | 0.0028 | 0.0028 | 0.8729 | 0.0526 | 0.4569 |
|  |  |  | [eT] x [eCO_2_] | 1 | 0.0056 | 0.0056 | 1.7448 | 0.1052 | 0.2198 |
|  |  |  | Residuals | 14 | 0.0446 | 0.0032 |  | 0.8437 |  |
|  |  |  | Total | 17 | 0.0529 |  |  | 1.0000 |  |
|  |  | Drought | [eTeCO_2_] | 1 | -0.0001 | -0.0001 | -0.0447 | -0.0019 | 0.9054 |
|  |  |  | [D] | 1 | 0.0207 | 0.0207 | 6.5374 | 0.2722 | **0.0095** |
|  |  |  | [eT x eCO_2_] x [D] | 1 | 0.0048 | 0.0048 | 1.5224 | 0.0634 | 0.2298 |
|  |  |  | Residuals | 16 | 0.0508 | 0.0032 |  | 0.6663 |  |
|  |  |  | Total | 19 | 0.0762 |  |  | 1.0000 |  |
|  | Gene | eT vs eCO_2_ | [eT] | 1 | 0.0043 | 0.0043 | 4.0633 | 0.2251 | **0.0215** |
|  |  |  | [eCO_2_] | 1 | -0.0001 | -0.0001 | -0.0569 | -0.0032 | 0.8781 |
|  |  |  | [eT] x [eCO_2_] | 1 | 0.0000 | 0.0000 | 0.0469 | 0.0026 | 0.8384 |
|  |  |  | Residuals | 14 | 0.0147 | 0.0011 |  | 0.7755 |  |
|  |  |  | Total | 17 | 0.0190 |  |  | 1.0000 |  |
|  |  | Drought | [eTeCO_2_] | 1 | 0.0043 | 0.0043 | 5.5215 | 0.1999 | **0.0226** |
|  |  |  | [D] | 1 | 0.0042 | 0.0042 | 5.3416 | 0.1934 | **0.0251** |
|  |  |  | [eT x eCO_2_] x [D] | 1 | 0.0006 | 0.0006 | 0.7552 | 0.0273 | 0.4809 |
|  |  |  | Residuals | 16 | 0.0125 | 0.0008 |  | 0.5793 |  |
|  |  |  | Total | 19 | 0.0215 |  |  | 1.0000 |  |
