# Supplementary material for: Composition and activity of nitrifier communities in soil are unresponsive to elevated temperature and CO2, but strongly affected by drought
Source: ISME J. 2020 Aug 7;14(12):3038–53. doi: 10.1038/s41396-020-00735-7 (PMC7784676; doi:10.1038/s41396-020-00735-7)
Supplement: Supplementary file 6 — Figure S1 [file 41396_2020_735_MOESM6_ESM.pdf]

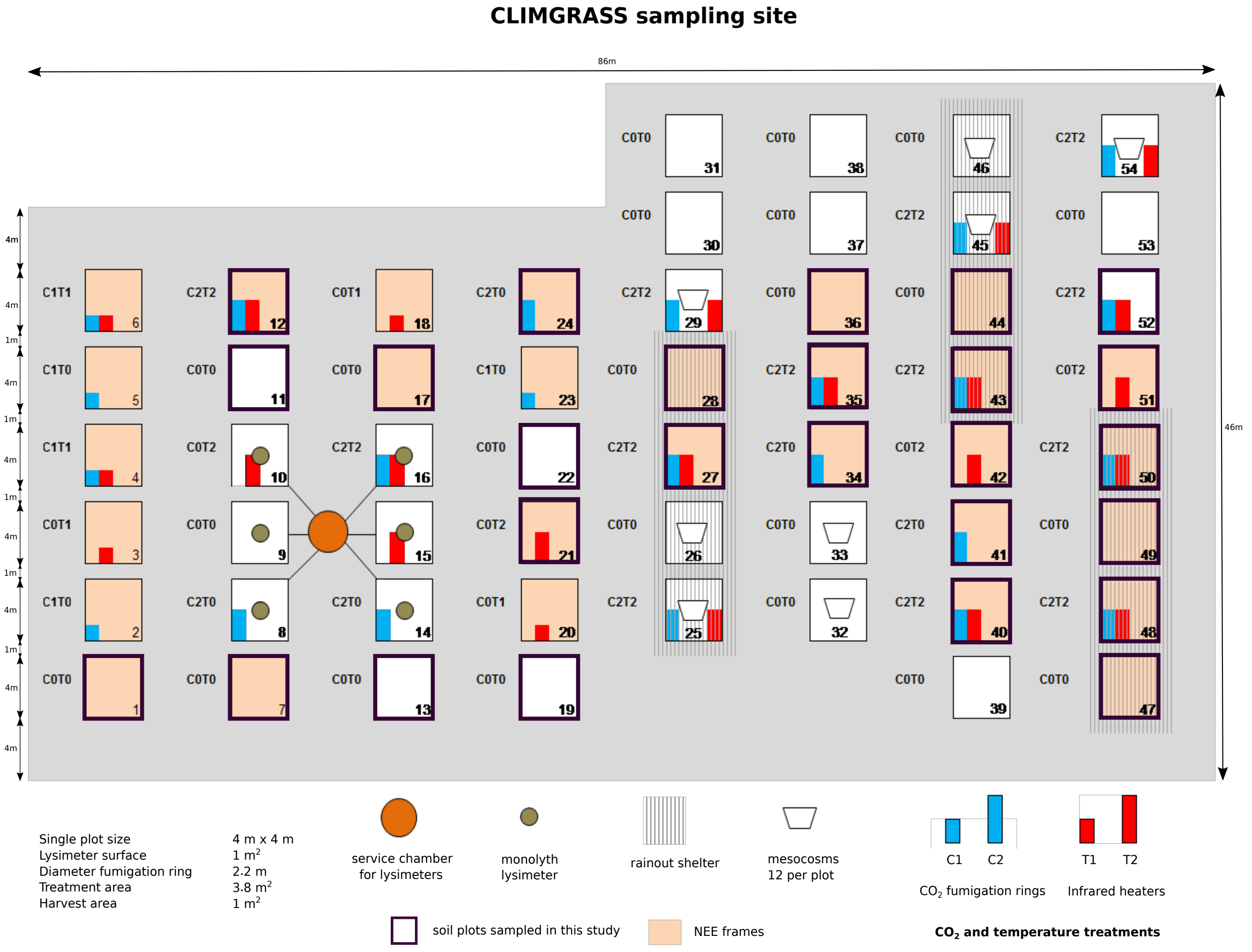


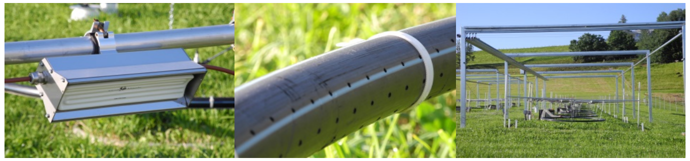

Infrared-heater Fumigation ring Rainout-shelter

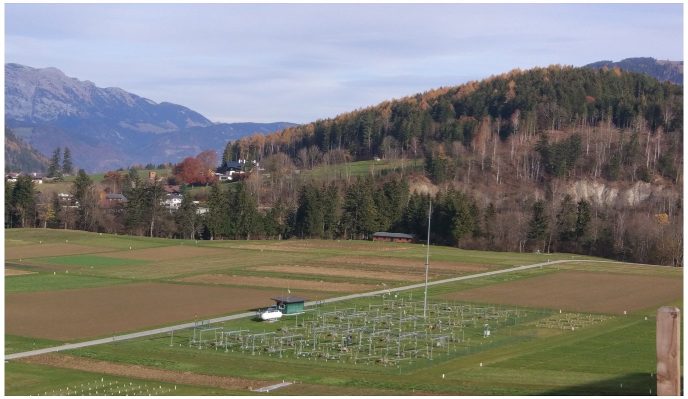

View of the field site. Photo credits: Joana Séneca

## Summary of plots sampled in this study

| Plot code | Treatment conditions | Plot number |
|---|---|---|
| c0t0 | [Amb] | 1,7,11,13,17,19,22,36 |
| c0t2 | [eT] | 21,42,51 |
| c2t0 | $eCO_2$ | 24,34,41 |
| c2t2 | [eT] x [$eCO_2$] | 12,35,40,52 |
| c0t0 | [Amb] x [D] | 28,44,47,49 |
| c2t2 | [$eTeCO_2$] x [D] | 27,43,48,50 |

**Figure S1:** Spatial arrangement of all plots from the Climgrass experimental site at HBLFA-Raumberg-Gumpenstein. The interdisciplinary teams involved in this project aim to monitor nutrient dynamics and changes on the hydrological, carbon and nitrogen cycles in montane grasslands, within a climate change scenario. For this experimental site, a completely randomized response surface design was used, which allows a more efficient estimation of treatment effects due to a more parsimonious modelling (Piepho et al. 2017). Rainout shelters were used to simulate drought, $CO_2$ fumigation rings increased atmospheric $CO_2$ levels by 150 ppm (C1) and 300 ppm (C2) above ambient conditions. Six infrared heaters per plot increased the soil temperature by 1.5 °C (T1) and 3 °C (T2) above ambient temperature. Out of 54 plots, 26 were sampled and analysed in this study. Sampled soil plots comprised the highest level of elevated temperature (+ 3 °C) and atmospheric $CO_2$ (+ 300 ppm) manipulations alone or in combination. Additionally, soil plots with rain-out shelters from ambient and from the most extreme combination of temperature and $CO_2$ were also sampled. More details regarding the sampling site and the maintenance of the facility are described in the Material and Methods section and on the website below.

Copyrights: HBLFA-Raumberg Gumpenstein. Figure availability: https://climgrass.at/experimental-site.html
