# Supplementary material for: Composition and activity of nitrifier communities in soil are unresponsive to elevated temperature and CO2, but strongly affected by drought
Source: ISME J. 2020 Aug 7;14(12):3038–53. doi: 10.1038/s41396-020-00735-7 (PMC7784676; doi:10.1038/s41396-020-00735-7)
Supplement: Supplementary file 7 — Figure S2 [file 41396_2020_735_MOESM7_ESM.pdf]

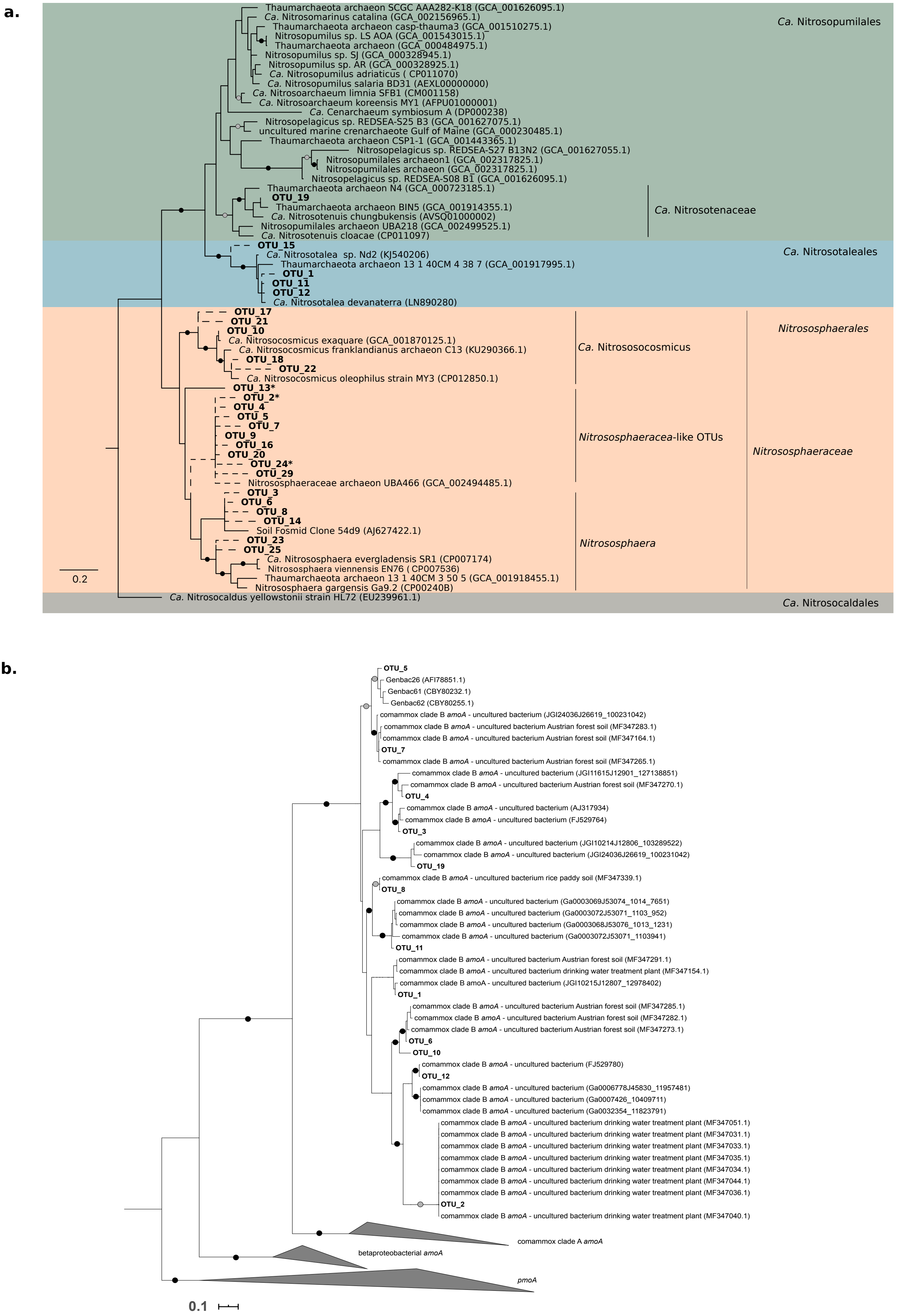
a.
Thaumarchaeota archaeon SCGC AAA282-K18 (GCA_001626095.1)
Ca. Nitrosomarinus catalina (GCA_002156965.1)
Thaumarchaeota archaeon casp-thauma3 (GCA_001510275.1)
Nitrosopumilus sp. LS AOA (GCA_001543015.1)
Thaumarchaeota archaeon (GCA_000484975.1)
Nitrosopumilus sp. SJ (GCA_000328945.1)
Nitrosopumilus sp. AR (GCA_000328925.1)
Ca. Nitrosopumilus adriaticus (CP011070)
Ca. Nitrosopumilus salaria BD31 (AEXL00000000)
Ca. Nitrosoarchaeum limnia SFB1 (CM001158)
Ca. Nitrosoarchaeum koreensis MY1 (AFPU01000001)
Ca. Cenarchaeum symbiosum A (DP000238)
Nitrosopelagicus sp. REDSEA-S25 B3 (GCA_001627075.1)
uncultured marine crenarchaeote Gulf of Maine (GCA_000230485.1)
Thaumarchaeota archaeon CSP1-1 (GCA_001443365.1)
Nitrosopelagicus sp. REDSEA-S27 B13N2 (GCA_001627055.1)
Nitrosopumilales archaeon1 (GCA_002317825.1)
Nitrosopumilales archaeon (GCA_002317825.1)
Nitrosopelagicus sp. REDSEA-S08 B1 (GCA_001626095.1)
Thaumarchaeota archaeon N4 (GCA_000723185.1)
OTU_19
Thaumarchaeota archaeon BIN5 (GCA_001914355.1)
Ca. Nitrosotenuis chungbukensis (AVSQ01000002)
Nitrosopumilales archaeon UBA218 (GCA_002499525.1)
Ca. Nitrosotenuis cloacae (CP011097)
Ca. Nitrosopumilales
Ca. Nitrosotenaceae
OTU_15
Ca. Nitrosotalea sp. Nd2 (KJ540206)
Thaumarchaeota archaeon 13 1 40CM 4 38 7 (GCA_001917995.1)
OTU_1
OTU_11
OTU_12
Ca. Nitrosotalea devanaterra (LN890280)
Ca. Nitrosotaleales
OTU_17
OTU_21
OTU_10
Ca. Nitrosocosmicus exaquare (GCA_001870125.1)
Ca. Nitrosocosmicus franklandianus archaeon C13 (KU290366.1)
OTU_18
OTU_22
Ca. Nitrosocosmicus oleophilus strain MY3 (CP012850.1)
Ca. Nitrososocosmicus
Nitrososphaerales
OTU_13*
OTU_2*
OTU_4
OTU_5
OTU_7
OTU_9
OTU_16
OTU_20
OTU_24*
OTU_29
Nitrososphaeraceae archaeon UBA466 (GCA_002494485.1)
Nitrososphaeracea-like OTUs
Nitrososphaeraceae
OTU_3
OTU_6
OTU_8
OTU_14
Soil Fosmid Clone 54d9 (AJ627422.1)
OTU_23
OTU_25
Ca. Nitrososphaera evergladensis SR1 (CP007174)
Nitrososphaera viennensis EN76 (CP007536)
Thaumarchaeota archaeon 13 1 40CM 3 50 5 (GCA_001918455.1)
Nitrososphaera gargensis Ga9.2 (CP00240B)
Nitrososphaera
0.2
Ca. Nitrosocaldus yellowstonii strain HL72 (EU239961.1)
Ca. Nitrosocaldales
b.
OTU_5
Genbac26 (AFI78851.1)
Genbac61 (CBY80232.1)
Genbac62 (CBY80255.1)
comammox clade B amoA - uncultured bacterium (JGI24036J26619_100231042)
comammox clade B amoA - uncultured bacterium Austrian forest soil (MF347283.1)
comammox clade B amoA - uncultured bacterium Austrian forest soil (MF347164.1)
OTU_7
comammox clade B amoA - uncultured bacterium Austrian forest soil (MF347265.1)
comammox clade B amoA - uncultured bacterium (JGI11615J12901_127138851)
comammox clade B amoA - uncultured bacterium Austrian forest soil (MF347270.1)
OTU_4
comammox clade B amoA - uncultured bacterium (AJ317934)
comammox clade B amoA - uncultured bacterium (FJ529764)
OTU_3
comammox clade B amoA - uncultured bacterium (JGI10214J12806_103289522)
comammox clade B amoA - uncultured bacterium (JGI24036J26619_100231042)
OTU_19
comammox clade B amoA - uncultured bacterium rice paddy soil (MF347339.1)
OTU_8
comammox clade B amoA - uncultured bacterium (Ga0003069J53074_1014_7651)
comammox clade B amoA - uncultured bacterium (Ga0003072J53071_1103_952)
comammox clade B amoA - uncultured bacterium (Ga0003068J53076_1013_1231)
comammox clade B amoA - uncultured bacterium (Ga0003072J53071_1103941)
OTU_11
comammox clade B amoA - uncultured bacterium Austrian forest soil (MF347291.1)
comammox clade B amoA - uncultured bacterium drinking water treatment plant (MF347154.1)
comammox clade B amoA - uncultured bacterium (JGI10215J12807_12978402)
OTU_1
comammox clade B amoA - uncultured bacterium Austrian forest soil (MF347285.1)
comammox clade B amoA - uncultured bacterium Austrian forest soil (MF347282.1)
comammox clade B amoA - uncultured bacterium Austrian forest soil (MF347273.1)
OTU_6
OTU_10
comammox clade B amoA - uncultured bacterium (FJ529780)
OTU_12
comammox clade B amoA - uncultured bacterium (Ga0006778J45830_11957481)
comammox clade B amoA - uncultured bacterium (Ga0007426_10409711)
comammox clade B amoA - uncultured bacterium (Ga0032354_11823791)
comammox clade B amoA - uncultured bacterium drinking water treatment plant (MF347051.1)
comammox clade B amoA - uncultured bacterium drinking water treatment plant (MF347031.1)
comammox clade B amoA - uncultured bacterium drinking water treatment plant (MF347033.1)
comammox clade B amoA - uncultured bacterium drinking water treatment plant (MF347035.1)
comammox clade B amoA - uncultured bacterium drinking water treatment plant (MF347034.1)
comammox clade B amoA - uncultured bacterium drinking water treatment plant (MF347044.1)
comammox clade B amoA - uncultured bacterium drinking water treatment plant (MF347036.1)
OTU_2
comammox clade B amoA - uncultured bacterium drinking water treatment plant (MF347040.1)
comammox clade A amoA
betaproteobacterial amoA
pmoA
0.1

Figure S2: Rooted maximum likelihood phylogenetic tree of a) archaeal *amoA* and b) CMX *amoA* gene and transcript sequences. Inserted short reads are depicted in dashed lines and sequenced OTUs are shown in bold. Asterisks depict archaeal OTUs retrieved by sequencing, but not covered by the qPCR primer pair. The AOA maximum likelihood tree was constructed using RAxML v. 8.2.12 with 100 rapid bootstraps and GTRGAMMA model of nucleotide evolution and rooted on *Ca.* Nitrosocaldus yellowstonni. Query sequences were inserted using the EPA algorithm implemented in RAxML after manual inspection of the alignment. Black and grey circles depict support values above 95 and between 80-95 respectively. The CMX tree was constructed from an alignment of query and reference sequences using W-IQ-Tree with 1000 bootstrap iterations and ModelFinder to determine the best fitting base substitution model. The tree was rooted on particulate methane monooxygenase (*pmoA*) gene sequences. Sequence accession numbers are shown in parenthesis. For details regarding tree construction, see Material and Methods.
