# Supplementary material for: Composition and activity of nitrifier communities in soil are unresponsive to elevated temperature and CO2, but strongly affected by drought
Source: ISME J. 2020 Aug 7;14(12):3038–53. doi: 10.1038/s41396-020-00735-7 (PMC7784676; doi:10.1038/s41396-020-00735-7)
Supplement: Supplementary file 8 — Figure S3 [file 41396_2020_735_MOESM8_ESM.pdf]

a.

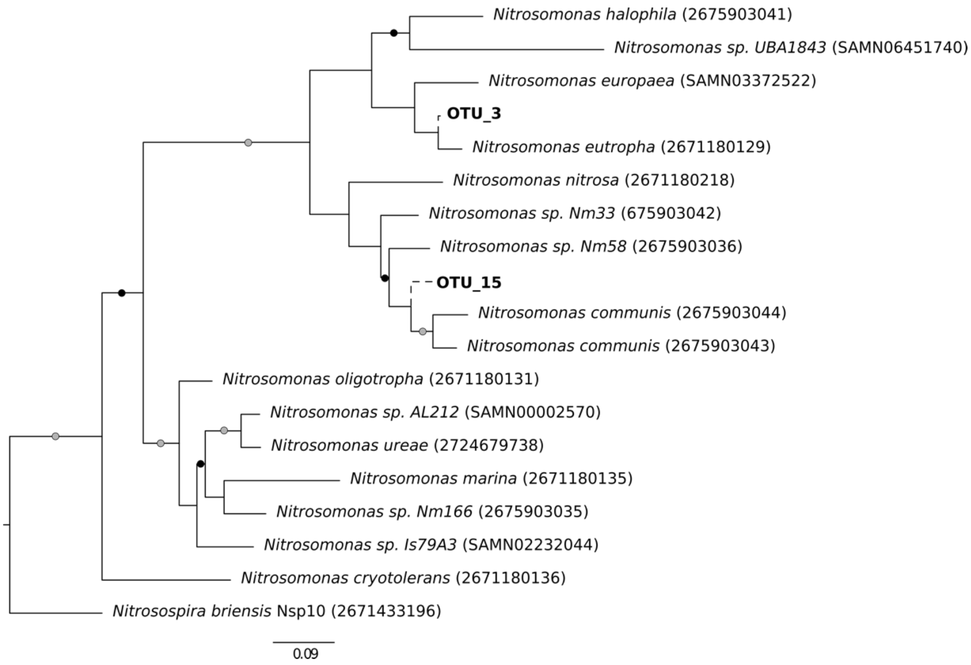


b.

| Query | Subject | Identity | E-value | Bitscore |
|---|---|---|---|---|
| OTU_1 | D19_AOB-968-133;size=1352 | 100 | 0 | 828 |
| OTU_2 | D12_AOB-26-1125;size=21 | 100 | 0 | 828 |
| OTU_4 | D16_AOB-75-722;size=8 | 100 | 0 | 828 |
| OTU_5 | D8_AOB-80-2013;size=13 | 100 | 0 | 828 |
| OTU_6 | D16_AOB-7-539;size=54 | 100 | 0 | 828 |
| OTU_9 | D21_AOB-7-796;size=16 | 100 | 0 | 828 |
| OTU_10 | D12_AOB-910-11289;size=5 | 91.071 | $2.23^{-174}$ | 606 |
| OTU_11 | D12_AOB-490-2083;size=3 | 95.759 | 0 | 723 |
| OTU_12 | D7_AOB-149-3286;size=12 | 98.214 | 0 | 784 |
| OTU_14 | D2_AOB-910-338;size=51 | 100 | 0 | 828 |
| OTU_24 | D9_Nitrosospira_sp._L115_(AY123817) | 99.517 | 0 | 754 |
| OTU_28 | D12_AOB-49-12128;size=2 | 93.304 | 0 | 662 |
| OTU_34 | D8_AOB-7-83;size=257 | 99.33 | 0 | 808 |

**Figure S3**: Taxonomic classification of bacterial *amoA* OTUs from genera a) *Nitrosospira* and b) *Nitrosomonas*. *Nitrosospira amoA* OTUs were classified by blastn against a reference database. Maximum likelihood phylogenetic tree of *Nitrosomonas amoA* gene sequences were constructed using RAxML v. 8.2.12 with 100 rapid bootstraps and GTRGAMMA model of nucleotide evolution. Query sequences were inserted into the reference tree using the EPA algorithm implemented in RAxML after manual inspection of the alignment. Inserted short reads are depicted in dashed lines. The tree was rooted on *Nitrosospira briensis*. Black and grey circles depict support values above 95 and between 80-95 respectively. Sequence accession numbers are shown in parenthesis. For details regarding tree construction and OTU classification, see Material and Methods.
