# Supplementary material for: Composition and activity of nitrifier communities in soil are unresponsive to elevated temperature and CO2, but strongly affected by drought
Source: ISME J. 2020 Aug 7;14(12):3038–53. doi: 10.1038/s41396-020-00735-7 (PMC7784676; doi:10.1038/s41396-020-00735-7)
Supplement: Supplementary file 9 — Figure S4 [file 41396_2020_735_MOESM9_ESM.pdf]

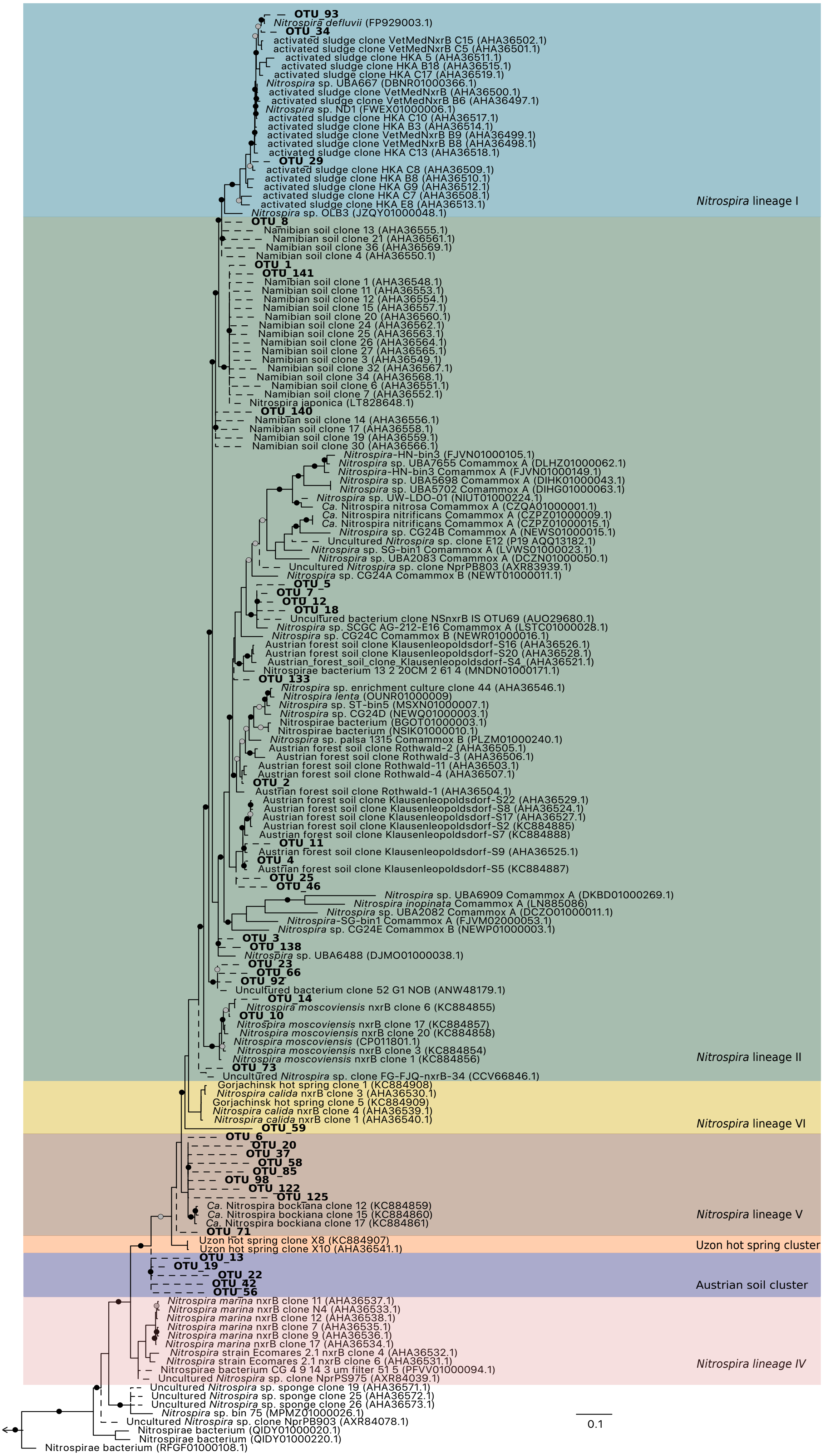

OTU_93
Nitrospira defluvii (FP929003.1)
OTU_34
activated sludge clone VetMedNxrB C15 (AHA36502.1)
activated sludge clone VetMedNxrB C5 (AHA36501.1)
activated sludge clone HKA 5 (AHA36511.1)
activated sludge clone HKA B18 (AHA36515.1)
activated sludge clone HKA C17 (AHA36519.1)
Nitrospira sp. UBA667 (DBNR01000366.1)
activated sludge clone VetMedNxrB (AHA36500.1)
activated sludge clone VetMedNxrB B6 (AHA36497.1)
Nitrospira sp. ND1 (FWEX01000006.1)
activated sludge clone HKA C10 (AHA36517.1)
activated sludge clone HKA B3 (AHA36514.1)
activated sludge clone VetMedNxrB B9 (AHA36499.1)
activated sludge clone VetMedNxrB B8 (AHA36498.1)
activated sludge clone HKA C13 (AHA36518.1)
OTU_29
activated sludge clone HKA C8 (AHA36509.1)
activated sludge clone HKA B8 (AHA36510.1)
activated sludge clone HKA G9 (AHA36512.1)
activated sludge clone HKA C7 (AHA36508.1)
activated sludge clone HKA E8 (AHA36513.1)
Nitrospira sp. OLB3 (JZQY01000048.1)
Nitrospira lineage I
OTU_8
Namibian soil clone 13 (AHA36555.1)
Namibian soil clone 21 (AHA36561.1)
Namibian soil clone 36 (AHA36569.1)
Namibian soil clone 4 (AHA36550.1)
OTU_1
OTU_141
Namibian soil clone 1 (AHA36548.1)
Namibian soil clone 11 (AHA36553.1)
Namibian soil clone 12 (AHA36554.1)
Namibian soil clone 15 (AHA36557.1)
Namibian soil clone 20 (AHA36560.1)
Namibian soil clone 24 (AHA36562.1)
Namibian soil clone 25 (AHA36563.1)
Namibian soil clone 26 (AHA36564.1)
Namibian soil clone 27 (AHA36565.1)
Namibian soil clone 3 (AHA36549.1)
Namibian soil clone 32 (AHA36567.1)
Namibian soil clone 34 (AHA36568.1)
Namibian soil clone 6 (AHA36551.1)
Namibian soil clone 7 (AHA36552.1)
Nitrospira japonica (LT828648.1)
OTU_140
Namibian soil clone 14 (AHA36556.1)
Namibian soil clone 17 (AHA36558.1)
Namibian soil clone 19 (AHA36559.1)
Namibian soil clone 30 (AHA36566.1)
Nitrospira-HN-bin3 (FJVN01000105.1)
Nitrospira sp. UBA7655 Comammox A (DLHZ01000062.1)
Nitrospira-HN-bin3 Comammox A (FJVN01000149.1)
Nitrospira sp. UBA5698 Comammox A (DIHK01000043.1)
Nitrospira sp. UBA5702 Comammox A (DIHG01000063.1)
Nitrospira sp. UW-LDO-01 (NIUT01000224.1)
Ca. Nitrospira nitrosa Comammox A (CZQA01000001.1)
Ca. Nitrospira nitrificans Comammox A (CZPZ01000009.1)
Ca. Nitrospira nitrificans Comammox A (CZPZ01000015.1)
Nitrospira sp. CG24B Comammox A (NEWS01000015.1)
Uncultured Nitrospira sp. clone E12 (P19 AQQ13182.1)
Nitrospira sp. SG-bin1 Comammox A (LVWS01000023.1)
Nitrospira sp. UBA2083 Comammox A (DCZN01000050.1)
Uncultured Nitrospira sp. clone NprPB803 (AXR83939.1)
Nitrospira sp. CG24A Comammox B (NEWT01000011.1)
OTU_5
OTU_7
OTU_12
OTU_18
Uncultured bacterium clone NSnxrB IS OTU69 (AUO29680.1)
Nitrospira sp. SCGC AG-212-E16 Comammox A (LSTC01000028.1)
Nitrospira sp. CG24C Comammox B (NEWR01000016.1)
Austrian forest soil clone Klausenleopoldsdorf-S16 (AHA36526.1)
Austrian forest soil clone Klausenleopoldsdorf-S20 (AHA36528.1)
Austrian_forest_soil_clone_Klausenleopoldsdorf-S4_(AHA36521.1)
Nitrospirae bacterium 13 2 20CM 2 61 4 (MNDN01000171.1)
OTU_133
Nitrospira sp. enrichment culture clone 44 (AHA36546.1)
Nitrospira lenta (OUNR01000009)
Nitrospira sp. ST-bin5 (MSXN01000007.1)
Nitrospira sp. CG24D (NEWQ01000003.1)
Nitrospirae bacterium (BGOT01000003.1)
Nitrospirae bacterium (NSIK01000010.1)
Nitrospira sp. palsa 1315 Comammox B (PLZM01000240.1)
Austrian forest soil clone Rothwald-2 (AHA36505.1)
Austrian forest soil clone Rothwald-3 (AHA36506.1)
Austrian forest soil clone Rothwald-11 (AHA36503.1)
Austrian forest soil clone Rothwald-4 (AHA36507.1)
OTU_2
Austrian forest soil clone Rothwald-1 (AHA36504.1)
Austrian forest soil clone Klausenleopoldsdorf-S22 (AHA36529.1)
Austrian forest soil clone Klausenleopoldsdorf-S8 (AHA36524.1)
Austrian forest soil clone Klausenleopoldsdorf-S17 (AHA36527.1)
Austrian forest soil clone Klausenleopoldsdorf-S2 (KC884885)
Austrian forest soil clone Klausenleopoldsdorf-S7 (KC884888)
OTU_11
Austrian forest soil clone Klausenleopoldsdorf-S9 (AHA36525.1)
OTU_4
Austrian forest soil clone Klausenleopoldsdorf-S5 (KC884887)
OTU_25
OTU_46
Nitrospira sp. UBA6909 Comammox A (DKBD01000269.1)
Nitrospira inopinata Comammox A (LN885086)
Nitrospira sp. UBA2082 Comammox A (DCZO01000011.1)
Nitrospira-SG-bin1 Comammox A (FJVM02000053.1)
Nitrospira sp. CG24E Comammox B (NEWP01000003.1)
OTU_3
OTU_138
Nitrospira sp. UBA6488 (DJMO01000038.1)
OTU_23
OTU_66
OTU_92
Uncultured bacterium clone 52 G1 NOB (ANW48179.1)
OTU_14
Nitrospira moscoviensis nxrB clone 6 (KC884855)
OTU_10
Nitrospira moscoviensis nxrB clone 17 (KC884857)
Nitrospira moscoviensis nxrB clone 20 (KC884858)
Nitrospira moscoviensis (CP011801.1)
Nitrospira moscoviensis nxrB clone 3 (KC884854)
Nitrospira moscoviensis nxrB clone 1 (KC884856)
OTU_73
Uncultured Nitrospira sp. clone FG-FJQ-nxrB-34 (CCV66846.1)
Nitrospira lineage II
Gorjachinsk hot spring clone 1 (KC884908)
Nitrospira calida nxrB clone 3 (AHA36530.1)
Gorjachinsk hot spring clone 5 (KC884909)
Nitrospira calida nxrB clone 4 (AHA36539.1)
Nitrospira calida nxrB clone 1 (AHA36540.1)
OTU_59
Nitrospira lineage VI
OTU_6
OTU_20
OTU_37
OTU_58
OTU_85
OTU_98
OTU_122
OTU_125
Ca. Nitrospira bockiana clone 12 (KC884859)
Ca. Nitrospira bockiana clone 15 (KC884860)
Ca. Nitrospira bockiana clone 17 (KC884861)
OTU_71
Nitrospira lineage V
Uzon hot spring clone X8 (KC884907)
Uzon hot spring clone X10 (AHA36541.1)
Uzon hot spring cluster
OTU_13
OTU_19
OTU_22
OTU_42
OTU_56
Austrian soil cluster
Nitrospira marina nxrB clone 11 (AHA36537.1)
Nitrospira marina nxrB clone N4 (AHA36533.1)
Nitrospira marina nxrB clone 12 (AHA36538.1)
Nitrospira marina nxrB clone 7 (AHA36535.1)
Nitrospira marina nxrB clone 9 (AHA36536.1)
Nitrospira marina nxrB clone 17 (AHA36534.1)
Nitrospira strain Ecomares 2.1 nxrB clone 4 (AHA36532.1)
Nitrospira strain Ecomares 2.1 nxrB clone 6 (AHA36531.1)
Nitrospirae bacterium CG 4 9 14 3 um filter 51 5 (PFVV01000094.1)
Uncultured Nitrospira sp. clone NprPS975 (AXR84039.1)
Nitrospira lineage IV
Uncultured Nitrospira sp. sponge clone 19 (AHA36571.1)
Uncultured Nitrospira sp. sponge clone 25 (AHA36572.1)
Uncultured Nitrospira sp. sponge clone 26 (AHA36573.1)
Nitrospira sp. bin 75 (MPMZ01000026.1)
Uncultured Nitrospira sp. clone NprPB903 (AXR84078.1)
Nitrospirae bacterium (QIDY01000020.1)
Nitrospirae bacterium (QIDY01000220.1)
Nitrospirae bacterium (RFGF01000108.1)
0.1

Figure S4. Maximum likelihood phylogenetic tree of *nxrB* sequences (NOB) constructed using RAxML v. 8.2.12 with 100 rapid bootstraps and GTRGAMMA model of nucleotide evolution. Query sequences were inserted into the reference tree using the EPA algorithm implemented in RAxML after manual inspection of the alignment. Inserted short reads are depicted in dashed lines and sequenced OTUs are shown in bold. The tree was rooted on the anammox bacterium *Kuenenia stuttgartiensis*. Black and grey circles depict support values above 95 and between 80-95 respectively. Sequence accession numbers are shown in parenthesis
