# Supplementary material for: Composition and activity of nitrifier communities in soil are unresponsive to elevated temperature and CO2, but strongly affected by drought
Source: ISME J. 2020 Aug 7;14(12):3038–53. doi: 10.1038/s41396-020-00735-7 (PMC7784676; doi:10.1038/s41396-020-00735-7)
Supplement: Supplementary file 10 — Figure S5 [file 41396_2020_735_MOESM10_ESM.pdf]

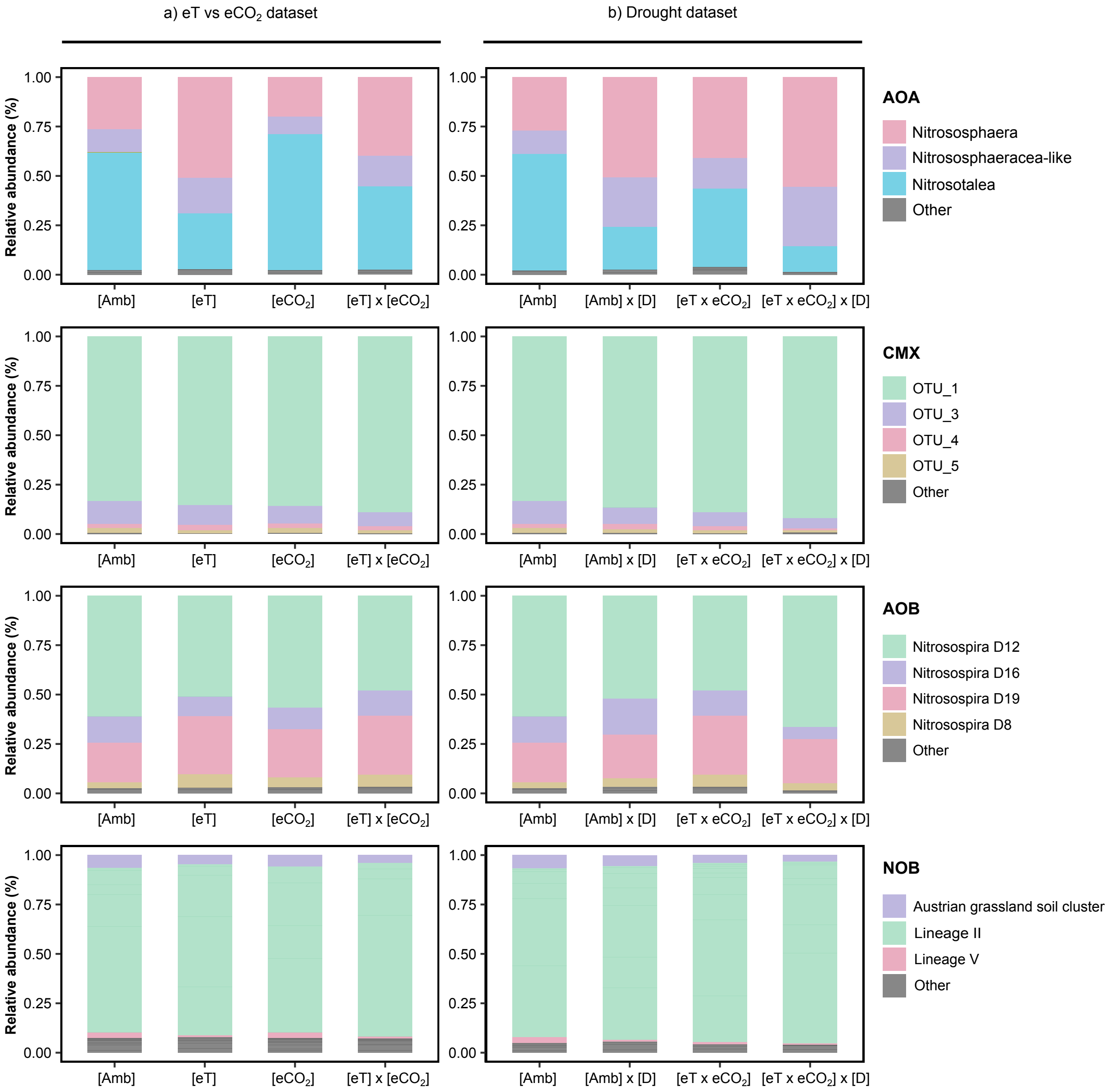


**Figure S5.** Relative abundance (%) of all nitrifying groups obtained by *amoA* and *nxrB* gene sequencing in plots with single and interactive effects of elevated temperature (eT) and atmospheric $CO_2$ ($eCO_2$) concentration – (A); and plots with single and interactive effects of future climate conditions (eT x $eCO_2$) and drought (D) – (B). The color code represents different genera/OTUs/clades/lineages. Multiple horizontal lines within the same color represent individual OTUs within a group.Taxa that comprised less than 0.1% of all reads per treatment are grouped as ‘Other’.
